# Granulins rescue inflammation, lysosome dysfunction, lipofuscin, and neuropathology in a mouse model of progranulin deficiency

**DOI:** 10.1016/j.celrep.2024.114985

**Published:** 2024-11-19

**Authors:** Jessica Root, Anarmaa Mendsaikhan, Georgia Taylor, Paola Merino, Srijita Nandy, Minzheng Wang, Ludmilla Troiano Araujo, Danny Ryu, Christopher Holler, Bonne M. Thompson, Giuseppe Astarita, Jean-François Blain, Thomas Kukar

**Affiliations:** 1 Department of Pharmacology and Chemical Biology, Emory University, School of Medicine, Atlanta, GA 30322, USA; 2 Center for Neurodegenerative Disease, Emory University, School of Medicine, Atlanta, GA 30322, USA; 3 Arkuda Therapeutics, 200 Arsenal Yards Blvd., Suite 220, Watertown, MA 02472, USA; 4 Department of Neurology, Emory University, School of Medicine, Atlanta, GA 30322, USA; 5 These authors contributed equally; 6 Lead contact

## Abstract

Progranulin (PGRN) deficiency is linked to neurodegenerative diseases, including frontotemporal dementia (FTD), Alzheimer’s disease, and Parkinson’s disease. Proper PGRN levels are critical for brain health; however, the function of PGRN is unclear. PGRN is composed of 7.5 repeat domains, called granulins, and processed into granulins inside the lysosome. PGRN is beneficial for neuronal health, but the role of individual granulins is controversial and unclear. We find that the expression of single granulins broadly rescues disease pathology in *Grn*^−/−^ mice. Adeno-associated virus (AAV)-mediated expression of human granulin-2/F, granulin-4/A, or PGRN in *Grn*^−/−^ mouse brain ameliorates dysregulated lysosomal proteins and lipids, microgliosis, and lipofuscinosis. Mechanistically, granulins localize to lysosomes in *Grn*^−/−^ mouse brains or fibroblasts. These data support the hypothesis that PGRN is a precursor to granulins, which share a beneficial function inside the lysosome to maintain lipid and protein homeostasis to prevent neurodegeneration. Thus, granulins are potential therapeutics to treat FTD-*GRN* and related diseases.

## In brief

Root et al. report that expression of individual granulins, 6 kDa subunits of progranulin (88 kDa), is sufficient to prevent disease-associated phenotypes in aged *Grn*^−/−^ mice. Granulins ameliorated lysosomal dysfunction, microglial activation, lipid dysregulation, and lipofuscin accumulation in *Grn*^−/−^ mouse brains, suggesting that granulins are the functional components of progranulin.

## Graphical Abstract

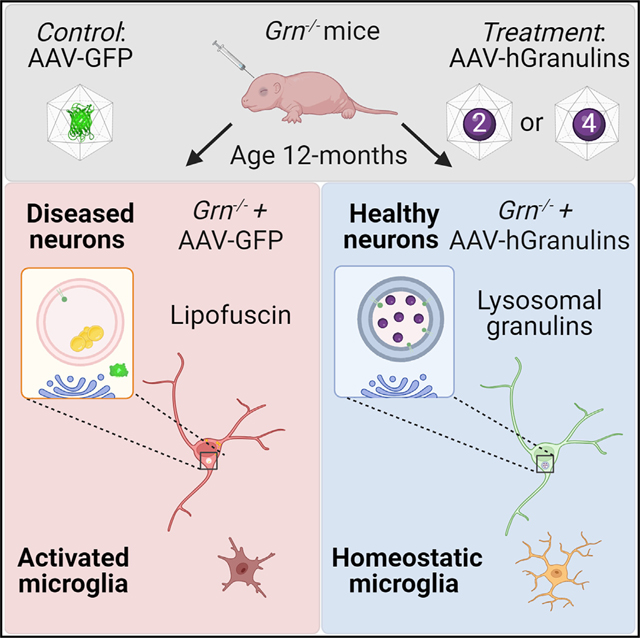

## INTRODUCTION

The granulin (*GRN*) gene encodes progranulin (PGRN), an evolutionarily conserved protein that is critical for neuronal survival.^1^ Haploinsufficiency of PGRN due to *GRN* mutations causes frontotemporal dementia (FTD), a common neurodegenerative disease in people under 60.^2–4^ Complete PGRN deficiency causes neuronal ceroid lipofuscinosis (NCL), a neurodegenerative lysosomal storage disorder (LSD).^5,6^ Moreover, genetic variants in *GRN* decrease PGRN and increase the risk of developing Alzheimer’s disease (AD), Parkinson’s disease (PD), or limbic-predominant age-related TDP-43 encephalopathy (LATE).^7–10^ Multiple therapies are being pursued to treat neurodegenerative diseases associated with decreased PGRN.^11,12^ Despite these advances, the function of PGRN is still unresolved and is a roadblock for drug development.

**Figure 1.**
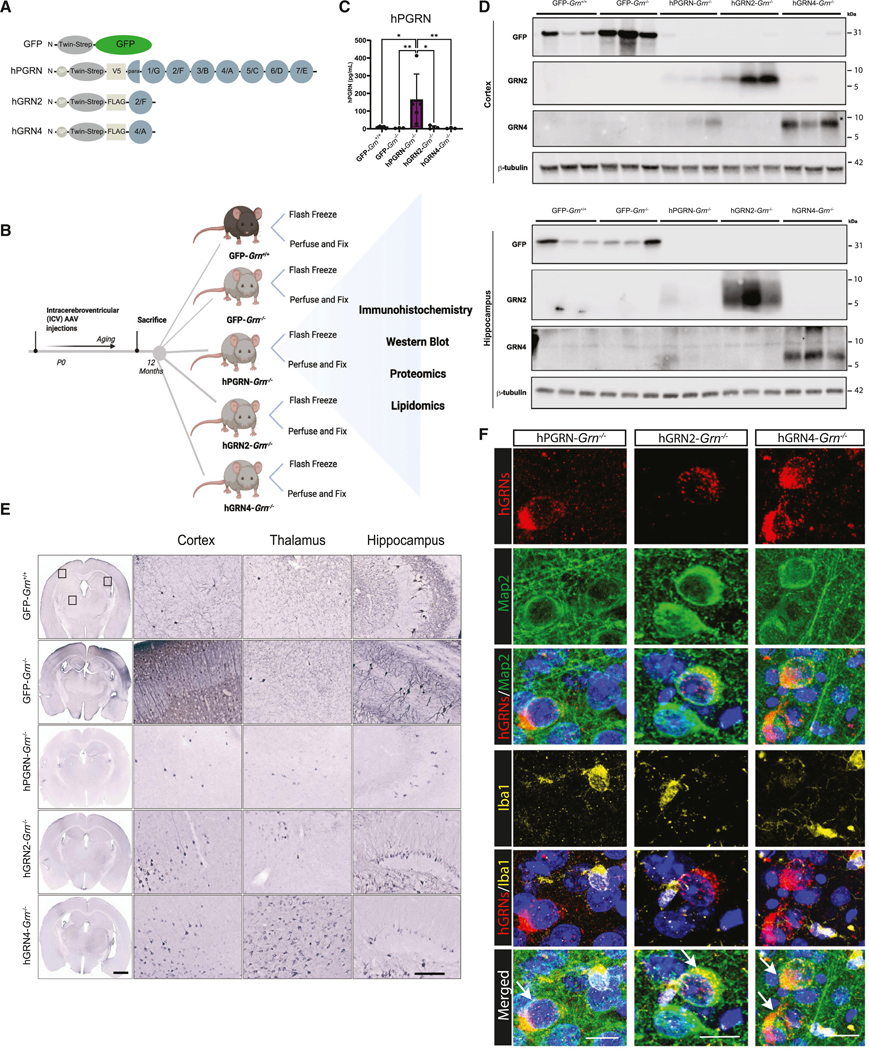
i.c.v. injection of rAAV at birth leads to expression of hGRN2, hGRN4, hPGRN, and GFP throughout the Grn^−/−^ mouse brain (A) Constructs including coding region, domains, and epitope tags that were packaged into rAAV2/1 (Twin-Strep tag; V5 tag; FLAG tag; SP, signal peptide; paragranulin; granulin-1 [GRN1; G]; GRN2 [F]; GRN3 [B]; GRN4 [A]; GRN5 [C]; GRN6 [D]; GRN7 [E]). (B) Experimental workflow includes intracerebroventricular (i.c.v.) injection of rAAV, mouse aging, sample collection, and sample analysis. (C) ELISA quantification of hPGRN in cortical tissue from rAAV-injected mice as mean ± SD. One-way ANOVA with Tukey’s post hoc correction. *n* = 6–7 mice/group. *p < 0.05 and **p < 0.01. (D) Immunoblot of cortical and hippocampal lysates verifying expression of GFP, hGRN2, and hGRN4 (β-tubulin loading control). (E) IHC images of Twin-Strep to visualize expression of GFP, hPGRN, hGRN2, and hGRN4 in coronal section plus magnified images of the cortex, hippocampus, and thalamus. Scale bars: 2 μm (full section) and 200 μm (magnified boxes). (F) Immunofluorescence (IF) images co-staining for hPGRN, hGRN2, and hGRN4 and antibody markers for neurons (Map2) and microglia (Iba1) in the cortex of an hPGRN, hGRN2-*Grn*^−/−^, and hGRN4-*Grn*^−/−^ mouse. Scale bar: 10 μm.

PGRN is a ~80 kDa glycoprotein that is ubiquitously expressed and enriched in microglia and neurons in the brain.^13–15^ Mammalian PGRN is composed of 7.5 tandem repeat proteins, called granulins (GRNs), that are stabilized by disulfide bonds (Figure 1A).^16^ Within PGRN, each GRN is joined together by short linkers, which can be cleaved by proteases to release mature GRNs.^14,17,18^ We refer to each GRN numbered 1 through 7 based on the UniProtKB database:P28799 rather than the colloquial A through G nomenclature. The relationship between the activity of PGRN and individual GRNs is debated and still unclear. Multiple functions have been ascribed to PGRN, including cell growth, neurotrophic signaling, and anti-inflammatory activity. The pleiotropic activity of PGRN may occur through binding signaling receptors^4,19^; however, some PGRN-receptor interactions have not been replicated,^20–22^ raising the possibility of other mechanisms of action. Furthermore, the function of individual GRNs, also called epithelins, is controversial and unresolved. Depending on the model, GRNs have been reported to be neurotrophic,^23^ pro-inflammatory,^17^ neurotoxic,^24^ or to impair lysosome function.^25^

The discovery by our lab, and others, that GRNs are made constitutively inside lysosomes led us to reevaluate the functional relationship between PGRN and GRNs.^26–28^ Because the complete loss of GRNs in humans and mice causes an LSD, with more severe neurodegeneration than PGRN haploinsufficiency, we reasoned that GRNs have an intra-lysosomal function. This idea is supported by the function of other lysosomal proteins like saposins, which are generated from prosaposin.^29^ Here, we test the hypothesis that PGRN is a precursor to GRNs, which are the functional units that mediate lysosomal homeostasis and are neuroprotective. We used recombinant adeno-associated virus (rAAV2/1) to assess whether expression of individual GRNs in the brain of PGRN-deficient mice can correct disease-associated phenotypes. We find that neuronal expression of a single GRN ameliorates a range of phenotypes, including lysosomal dysfunction, microgliosis, lipid abnormalities, and lipofuscin accumulation, to the same extent as full-length PGRN. These findings provide compelling evidence that GRNs are the bioactive subunits of PGRN and indicate that potential therapeutic approaches for FTD-*GRN* should consider their effect on GRN levels. Furthermore, this work supports the potential use of GRNs for the treatment of diseases associated with PGRN deficiency.

## RESULTS

### Injection of rAAV at birth leads to widespread expression of GRNs, PGRN, and GFP throughout the mouse brain

We utilized *Grn*^−/−^ mice to test the hypothesis that GRNs are functionally active and neuroprotective. These mice lack PGRN and develop neuropathology similar to NCL and FTD-*GRN*, including neuroinflammation and microgliosis,^30–32^ alterations in lysosomal gene expression,^33,34^ and accumulation of lipofuscin.^35,36^ In these experiments, we compared human GRN2 (hGRN2) and hGRN4, as previous studies suggested they have opposing functional activity.^37^ Furthermore, hGRN2 (also known as GRN F) and hGRN4 (also known as GRN A) share 50% identity at the amino acid level, and we reasoned that this would be sufficient to reveal differences in bioactivity if present (Figures S1A and S1B). Human PGRN (hPGRN) and GFP served as positive and negative controls, respectively. The endogenous N-terminal signal peptide (SP) from hPGRN was engineered to precede hGRN2 and hGRN4 expression constructs to ensure trafficking through the secretory pathway, which is important for the folding and sorting of lysosomal proteins.^38^ This sequence was followed by epitope tags (Twin-Strep tag and V5 or FLAG) preceding the coding region to facilitate detection (Figure 1A). We verified our constructs by transfecting HeLa *GRN*^−/−^ cells and collected media and cell lysates (Figure S2). As expected, hPGRN is secreted into the media and accumulates as an ~80 kDa band (Figures S2A and S2B). hGRN2 and hGRN4 are secreted into the media as ~15 kDa proteins that are immunoreactive for Twin-Strep tag and hGRN2 and hGRN4 antibodies, respectively. In cell lysates, we observe full-length, unprocessed hGRN2 and hGRN4 (~15 kDa), as well as bands for cleaved hGRN2 (~6 kDa) and hGRN4 (~8 kDa) (Figure S2A). These bands retain immunoreactivity for anti-GRN antibodies but lose reactivity for the Twin-Strep tag, demonstrating that the tag has been cleaved. Moreover, the mature hGRN2 and hGRN4 bands migrate at the same molecular weight as endogenous GRNs in HeLa wild-type cells or HeLa *GRN*^−/−^ cells following re-expression of hPGRN. These data demonstrate that the hPGRN, hGRN2, and hGRN4 constructs are properly secreted as well as trafficked to the lysosome and cleaved into GRNs.^26^

Following *in vitro* validation, we generated hybrid rAAV2/1 encoding hGRN2, hGRN4, hPGRN, and GFP and performed bilateral intracerebroventricular (i.c.v.) injections of rAAV2/1 vectors into newly born (postnatal day P0) litters of *Grn*^−/−^ and *Grn*^+/+^ mice (Figure 1B; see Table S1). This experimental paradigm, termed somatic brain transgenesis (SBT), preferentially transduces neurons when using AAV vectors packaged in the capsid 1 serotype and leads to widespread and long-term expression of genes of interest in the mouse brain.^39–41^ All mice were aged to 12 months old, a time when substantial neuropathological changes occur in *Grn*^−/−^ mice. Next, we characterized the distribution and expression of each rAAV2/1 vector throughout the brain of injected *Grn*^−/−^ and *Grn*^+/+^ mice. To confirm the specific identity of expressed proteins, we performed immunoblotting of lysates from flash-frozen cortical and hippocampal brain tissue. Using specific GRN antibodies produced in our lab, we confirmed the expression of hGRN2 in hGRN2-*Grn*^−/−^ mice, hGRN4 in hGRN4-*Grn*^−/−^ mice, and GFP in GFP-*Grn*^−/−^ and *Grn*^+/+^ mice in both the hippocampus and cortex (Figure 1D). These antibodies recognize full-length hPGRN and GRNs in HeLa cell lysates and media. The selectivity and specificity of anti-GRN antibodies were confirmed using *GRN*^−/−^ cells (Figures S2A–S2C). Importantly, using these antibodies, we detect production of hGRN2 and hGRN4 in cortex and hippocampal lysates of AAV-hPGRN-injected *Grn*^−/−^ mice (Figures S3A and S3B). Full-length hPGRN was difficult to detect in mouse brain lysates by immunoblot, likely due to its rapid cleavage into GRNs.^26^ Instead, we used a commercial ELISA to confirm the expression of hPGRN in hPGRN-*Grn*^−/−^ mice (Figure 1C). Immunostaining of serial coronal sections for the Twin-Strep tag, which is shared across expression constructs, visualized and verified widespread expression of all encoded proteins in the hippocampus, thalamus, and cortex across injected mice (Figure 1E). Then, we assessed which cell types in the brain expressed GRNs following rAAV2/1 transduction. We performed immunofluorescent co-labeling of hPGRN, hGRN2, or hGRN4 with the neuronal marker Map2, the microglial marker Iba1 (Figure 1F), or the astrocytic marker Gfap (Figure S4A) in hPGRN-*Grn*^−/−^, hGRN2-*Grn*^−/−^, and hGRN4-*Grn*^−/−^ mice. We find that the vast majority of signals for hPGRN, hGRN2, or hGRN4 are found in cells expressing the neuronal marker Map2. We observed rare co-localization of hPGRN and hGRN4 in Iba1-positive cells (Figure S4B). In contrast, we did not observe any co-localization with Gfap-positive cells (Figure S4A). Thus, neonatal i.c.v. injection of rAAV2/1 produced robust and stable neuronal expression of transgenes in mouse brains over the 12 month period of our experiments.

### Proteome-wide dysregulation in the thalamus of *Grn*^−/−^ mice is ameliorated by expression of GRNs

The thalamus is a major site of pathologic changes in the brains of *Grn*^−/−^ mice^42^ and patients with FTD-*GRN*^43,44^; however, the underlying pathogenic mechanisms are still poorly defined. To provide deeper insight into dysfunction of the thalamus caused by PGRN deficiency, we performed quantitative proteomics on the thalamus from 12-month-old *Grn*^−/−^ mice injected with rAAV2/1 encoding hGRN2, hGRN4, hPGRN, or GFP and *Grn*^+/+^ mice injected with rAAV2/1 encoding GFP (Figure 1B). Thalamic lysates were labeled with isobaric tandem mass tags (TMTs), followed by offline electrostatic repulsion-hydrophilic interaction chromatography (ERLIC) fractionation prior to liquid chromatography-tandem mass spectrometry (LC-MS/MS), resulting in the identification and quantification of 9,255 proteins across all samples (Figure S5A; see Table S2).

**Figure 2.**
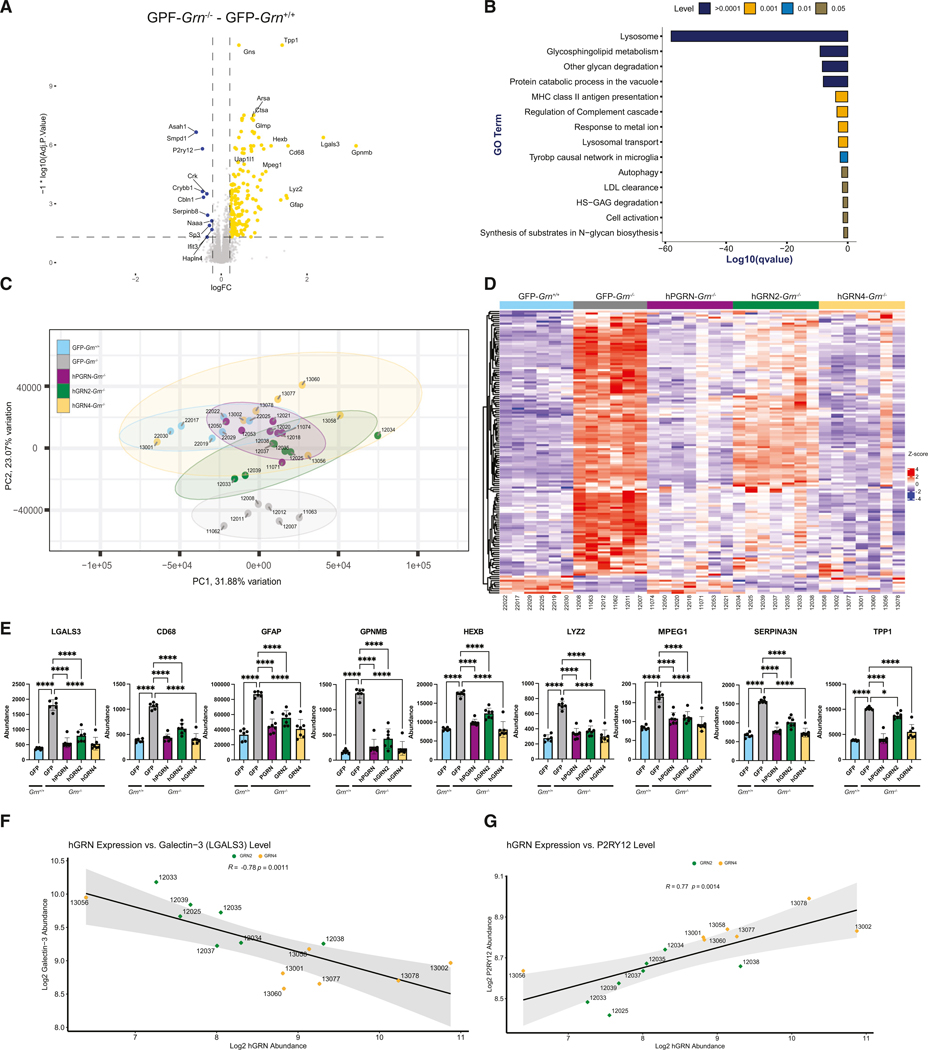
GRN2 and GRN4 prevent widespread protein dysregulation caused by PGRN deficiency in the thalamus of Grn^−/−^ mice (A) Volcano plot of upregulated (yellow) and downregulated proteins (blue) in the thalamus of GFP-*Grn*^−/−^ vs. GFP-*Grn*^+/+^ mice (fold change [FC] > 1.2, *p* < 0.05). (B) Bar graph of the most significantly enriched Gene Ontology (GO) terms describing the differentially expressed proteins in (A) (GFP-*Grn*^−/−^ mice vs. GFP-*Grn*^+/+^ mice; FC = 1.2 and adjusted *p* value = 0.05). Displaying all significant changed modules (*p* < 0.05). (C) Plot of PCs (PC1 vs. PC2) for GFP-*Grn*^+/+^ mice (blue), GFP-*Grn*^−/−^ mice (gray), hPGRN-*Grn*^−/−^ mice (purple), hGRN2-*Grn*^−/−^ mice (green), and hGRN4-GFP-*Grn*^−/−^ mice (yellow). Ellipses: 95% confidence interval. (D) Heatmap of top 140 proteins (rows) differentially expressed between GFP-*Grn*^−/−^ and GFP-*Grn*^+/+^ and treatment groups (columns). Quantification of individual proteins shown (log_2_*Z* score transformed). Individual mouse numbers are below the column. (E) Bar plots comparing correction of elevated levels of LGALS3, CD68, GFAP, GPNMB, HEXB, LYZ2, MPEG1, SERPINA3N, and TPP1 in *Grn*^−/−^ mice injected with GFP, hGRN2, hGRN4, or hPGRN. Mean (protein abundance) ± SD. One-way ANOVA with Tukey’s post hoc. *n* = 5–7 mice/group. *p < 0.05, **p < 0.01, ***p < 0.001, and ****p < 0.0001. (F) Correlation of hGRN2 (green) and hGRN4 (yellow) expression with galectin-3 (R = 0.78, *p* = 0.0011). (G) Correlation of hGRN2 (green) and hGRN4 (yellow) expression with P2RY12 (R = 0.77, *p* = 0.0014).

We next compared the GFP-*Grn*^+/+^ and GFP-*Grn*^−/−^ thalamic proteomes to identify differentially expressed proteins. In GFP-*Grn*^−/−^ mice, we identified 131 proteins that increased and 9 proteins that decreased in abundance in the thalamus compared to GFP-*Grn*^+/+^ mice (R1.2-fold change; false discovery rate [FDR] q < 0.05; Figure 2A; see Table S2). Gene Ontology (GO) analysis of the top 100 differentially expressed proteins using Metascape found a significant enrichment (−log10(*p*) > 10) of proteins involved in lysosome function (KEGG mmu04142), glycosphingolipid metabolism (R-MMU-1660662), and protein catabolic processes in the vacuole (GO: 0007039) (Figure 2B). Some of the most significantly dysregulated proteins in *Grn*^−/−^ mice included lysosomal hydrolases (ARSA, GNS, HEXA, HEXB, MANBA) and proteases (CTSD, DPP7, LGMN, TPP1). Additionally, modules related to inflammatory processes were significantly enriched (−log10(*p*) > 5), including major histocompatibility complex (MHC) class II antigen presentation (R-MMU-2132295) and regulation of the complement cascade (R-MMU-977606), which includes C1Qa, C1Qb, C1Qc, and C1Qb.

### Individual GRNs rescue dysregulated proteins in the thalamus of *Grn*^−/−^ mice

After characterizing differences between the proteomes of *Grn*^−/−^ and *Grn*^+/+^ mice, we asked if expression of GRNs or hPGRN ameliorated the changes observed in *Grn*^−/−^ thalamus. To visualize changes more easily between experimental groups, we used principal-component analysis (PCA) to reduce the complexity and dimensionality of the proteomic datasets. Using PCA, we extracted 10 components from the combined AAV-injected *Grn*^−/−^ and *Grn*^+/+^ proteomics dataset, which together account for 93% of variance in the samples (Figure S5B). Comparing principal components 1 and 2 (PC1 and PC2) revealed a clear separation of GFP-*Grn*^+/+^ (blue) and GFP-*Grn*^−/−^ (gray) samples, with no overlap between these two groups (Figure 2C). Samples from hGRN2-*Grn*^−/−^ (green), hGRN4-*Grn*^−/−^ (yellow), and hPGRN-*Grn*^−/−^ (purple) mice did overlap and cluster closer together with GFP-*Grn*^+/+^ mice, revealing a shift toward wild-type mice and away from *Grn*^−/−^ mice, suggesting a correction of altered protein levels.

To evaluate the rescue of disease-linked phenotypes in more detail, we created a heatmap containing the 140 differentially expressed proteins from the GFP-*Grn*^−/−^ and GFP-*Grn*^+/+^ proteomics comparison and included hGRN2-*Grn*^−/−^, hGRN4-*Grn*^−/−^, and hPGRN-*Grn*^−/−^ samples (Figure 2D). Visually, the groups of *Grn*^−/−^ mice treated with hGRN2, hGRN4, and PGRN are more like GFP-*Grn*^+/+^ than GFP-*Grn*^−/−^ mice. To provide a quantitative measurement of rescue, we compared the expression levels of the most upregulated (2-fold; *p* < 0.001) proteins (LGALS3, CD68, GFAP, GPNMB, HEXB, LYZ2, MPEG1, SERPINA3N, and TPP1) in the GFP-*Grn*^−/−^ proteome across rAAV treatment groups. rAAV-mediated expression of hGRN2, hGRN4, or hPGRN in *Grn*^−/−^ mice significantly decreased expression levels back toward wild-type levels of all nine proteins, indicating the correction of abnormally elevated proteins (Figure 2E). This analysis provides compelling evidence that expression of an individual GRN in the *Grn*^−/−^ mouse brain can functionally substitute the full-length PGRN protein.

Of note, in some cases, elevated proteins in *Grn*^−/−^ mouse brains were not corrected as efficiently in hGRN2-injected groups compared to hGRN4- and hPGRN-injected groups (e.g., TPP1; Figure 2E). One explanation for this result is that GRNs are expressed at different levels between groups. To investigate this, we compared expression levels of hGRN2 and hGRN4 in the *Grn*^−/−^ thalamic proteomics dataset by examining a tryptic fragment of the Twin-Strep-FLAG tag shared between both proteins, revealing that hGRN4 expression was ~2.5-fold higher than that of hGRN2 (Figure S5C). We then asked whether the expression level of GRN2 or GRN4 correlated with phenotypic rescue. Notably, the abundance of hGRN2 and hGRN4 correlated with galectin-3 (R = −0.78; *p* = 0.0011) (Figure 2F) and P2RY12 (R = 0.77 *p* = 0.0014) levels (Figure 2G) in the *Grn*^−/−^ thalamic proteome. Further, in individual mice, higher levels of either hGRN2 or hGRN4 correlated with the correction of altered protein levels, suggesting that the apparent decreased efficacy of hGRN2 is due to lower expression levels, not function. Taken together, proteomic analysis of the thalamus *of Grn*^−/−^ mouse reveals that rAAV-mediated expression of a single GRN ameliorates widespread protein dysregulation caused by loss of PGRN.

### Markers of lysosomal dysfunction are rescued by GRN expression across brain regions

**Figure 3.**
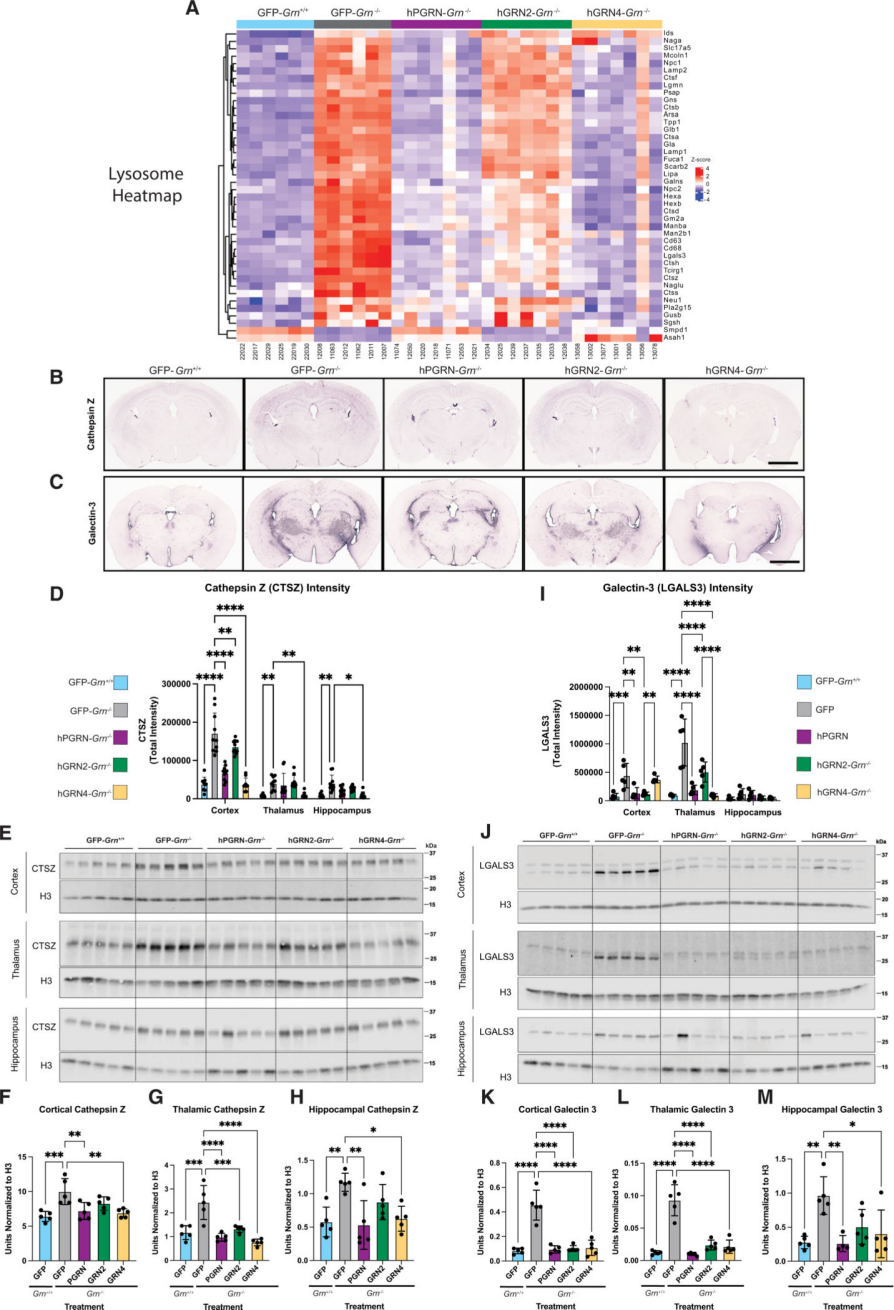
GRNs ameliorate dysregulated lysosomal proteins, including cathepsin Z and galectin-3, in the brains of Grn^−/−^ mice (A) Heatmap of differentially expressed (log_2_*Z* score transformed) lysosomal proteins (GO module) in GFP-*Grn*^−/−^ and GFP-*Grn*^+/+^ mice. 42 proteins are included (rows) across mice from all treatment groups (columns). (B and C) Representative (B) cathepsin Z and (C) galectin-3 IHC of coronal sections from rAAV-injected groups (GFP, hPGRN, hGRN2, hGRN4). Scale bar: 2 mm. (D) Quantification of cathepsin Z IHC signal in cortex, hippocampus, and thalamus. (E) Immunoblot for cathepsin Z in cortical, hippocampal, and thalamic brain lysates from all injection groups. (F–H) Quantification of (F) cortical, (G) thalamic, and (H) hippocampal immunoblot of cathepsin Z normalized to H3. (I) Quantification of galectin-3 IHC signal in cortex, hippocampus, and thalamus. (J) Immunoblot for galectin-3 in cortical, hippocampal, and thalamic brain lysates from all injection groups. (K–M) Quantification of (K) cortical, (L) thalamic, and (M) hippocampal galectin-3 immunoblot normalized to H3. Data are presented as means ± SD. *n* = 5 mice/group. *p* values were calculated by one-way or two-way (D and I) ANOVA with Tukey’s post hoc analysis. *p < 0.05, **p < 0.01, ***p < 0.001, and ****p < 0.0001.

To confirm and extend the proteomics data, we analyzed tissue from additional, independent cohorts of rAAV2/1-injected *Grn*^+/+^ and *Grn*^−/−^ mice that were processed for immunohistochemistry (IHC), immunoblot (western blot), or lipidomics (Figure 1B). In human patients, the complete loss of PGRN leads to a lysosomal storage disease called NCL. Characteristics of NCL have been identified in FTD-*GRN* mutation carriers, and many of these are recapitulated in *Grn*^−/−^ mice. These include signs of lysosomal dysfunction like the upregulation of lysosomal proteins, altered lysosomal enzyme activity, and disruption of macromolecule catabolism.^45^ Considering this, we expected that lysosomal dysfunction would be a major phenotype detected by our proteomic analysis, and “lysosome” was the most significant GO term in the GFP-*Grn*^−/−^ thalamic proteome. To understand which proteins from our dataset contributed to this GO term, we constructed a heatmap with all proteins that were assigned to this module (Figure 3A). To further validate these finding, we focused on two markers of lysosomal dysfunction: cathepsin Z (CTSZ)^42^ (Figures 3B and 3D–3H) and galectin-3 (LGALS3) (Figure 3C and 3I–3M). Cathepsin Z is a lysosomal cysteine protease that is upregulated in LSDs and neurodegenerative diseases.^46–48^ We performed IHC to examine the levels of cathepsin Z in hippocampal, thalamic, and cortical tissues of *Grn*^−/−^ mice injected with rAAV2/1 expressing GFP, hGRN2, hGRN4, and hPGRN (*n* = 5; one section/mouse) (Figure 3B). Quantification of IHC staining in the cortex, thalamus, and hippocampus was performed using CellProfiler (Figure S6) and revealed a significant increase in cathepsin Z signal in GFP-*Grn*^−/−^ mice across all regions examined (Figure 3D). Expression of hGRN2, hGRN4, and hPGRN corrected elevated levels of cathepsin Z in the cortex (Figure 3D). In the thalamus and hippocampus, only hGRN4 expression led to a statistically significant decrease in the level of cathepsin Z in these samples (Figure 3D).

We then performed immunoblotting on separate cohorts of mice to provide a complementary measurement of cathepsin Z in hippocampal, thalamic, and cortical tissue samples (Figure 3E). Cathepsin Z was increased in the GFP-*Grn*^−/−^ mouse cortex, hippocampus, and thalamus compared to wild-type counterparts (Figures 3E–3H). In agreement with the proteomics analyses, cathepsin Z levels were normalized by expression of hGRN2, hGRN4, and hPGRN in the *Grn*^−/−^ thalamus (Figures 3E and 3G). Cathepsin Z levels were also decreased in the cortex and hippocampus by hGRN4 and hPGRN, while hGRN2 treatment trended lower but did not reach significance (Figures 3F and 3H).

We also assessed levels of galectin-3 (LGALS3), a beta-galactoside binding lectin that is recruited to damaged lysosomes^49^ to facilitate lysosomal repair.^50^ Immunostaining of 12-month-old GFP-*Grn*^+/+^, GFP-*Grn*^−/−^, hPGRN-*Grn*^−/−^, hGRN2*-Grn*^−/−^, and hGRN4-*Grn*^−/−^ mouse coronal sections demonstrated that galectin-3 was increased in the thalamus and cortex of GFP-*Grn*^−/−^ mice (Figure 3C). Similarly to cathepsin Z, expression of hGRN2, hGRN4, and hPGRN corrected elevated galectin-3 in the thalamus compared to GFP-*Grn*^+/+^ mice (Figure 3I).

These results were further validated using immunoblot to measure galectin-3 levels in lysates of the cortex, thalamus, and hippocampus tissue from a separate cohort of rAAV2/1-injected mice (*n* = 5) (Figure 3J). We confirmed that galectin-3 was upregulated in cortical, thalamic, and hippocampus tissue lysates of GFP-*Grn*^−/−^ mice compared to GFP-*Grn*^+/+^ mice (Figures 3J–3M). Importantly, rAAV-mediated expression of hGRN2, hGRN4, and hPGRN reduced elevated galectin-3 expression in cortical and thalamic tissues (Figures 3K and 3L). In hippocampal samples, hGRN4 and hPGRN significantly reduced elevated galectin-3 levels in *Grn*^−/−^ mice (Figure 3M). In summary, we find that IHC and immunoblot analysis confirm and extend our proteomics data, demonstrating that expression of hGRN2 or hGRN4 ameliorates elevated cathepsin Z and galectin-3 levels throughout multiple brain regions of *Grn*^−/−^ mice. These data provide more evidence that a single GRN can functionally substitute the activity of full-length PGRN.

### Microglial activation and inflammatory markers are reduced by hGRNs

**Figure 4.**
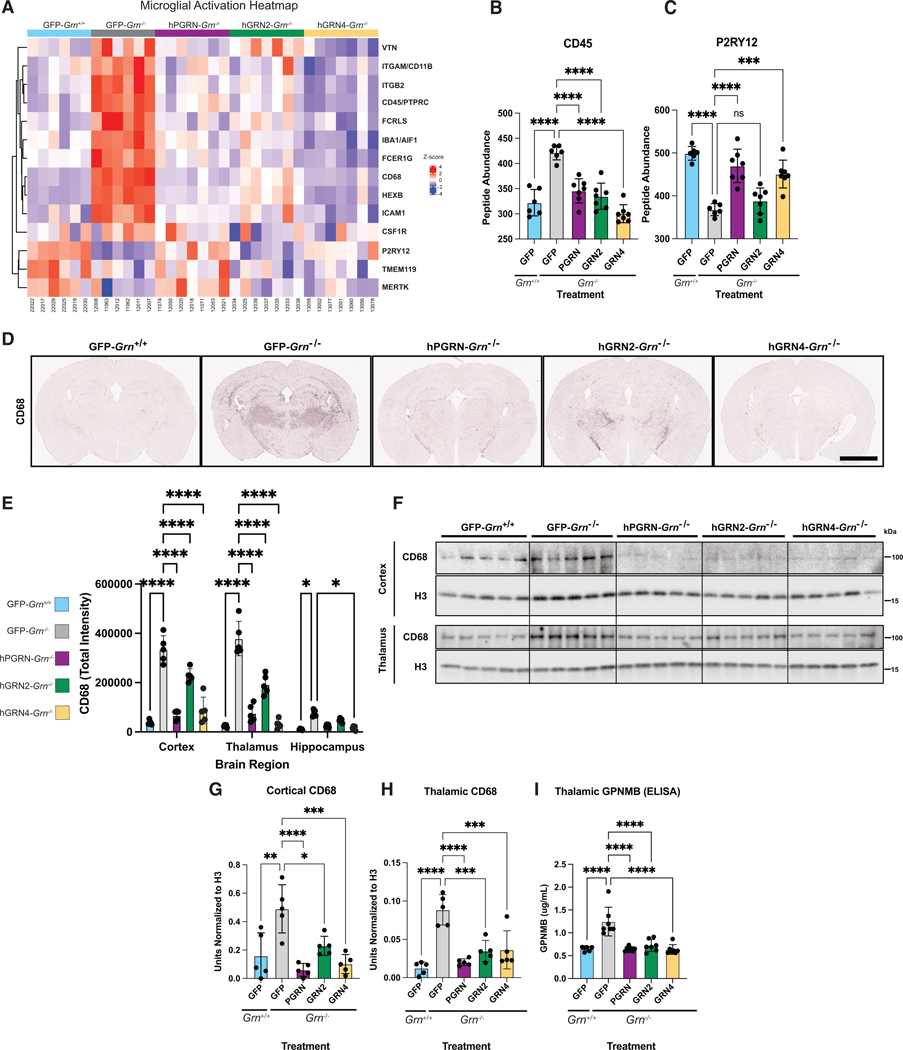
GRNs ameliorate markers of microglial activation in Grn^−/−^ mouse brains (A) Heatmap of differentially expressed (log_2_*Z* score-transformed) proteins associated with microglial activation and dysfunction (rows) in all treatment groups in GFP-*Grn*^−/−^ compared to GFP-*Grn*^+/+^ (columns). (B) Abundance of CD45 (PTPRC) across all treatment groups. (C) Abundance of P2RY12 across all treatment groups. (D) Representative CD68 IHC of 12-month-old mouse coronal brain sections across all treatment groups. Scale bar: 2 mm. (E) Quantification of CD68 IHC signal of cortex, hippocampus, and thalamus. (F) Immunoblot of CD68 in cortical and thalamic brain tissue from all injection groups. (G) Quantification of immunoblot of cortical CD68 signal normalized to H3. (H) Quantification of immunoblot of thalamic CD68 signal normalized to H3. (I) Quantification of GPNMB levels in thalamus using ELISA. Data are presented as means ± SD. *p* values were calculated by one-way or two-way (E) ANOVA with Tukey’s post hoc analysis. *p < 0.05, **p < 0.01, ***p < 0.001, and ****p < 0.0001.

While neurons express PGRN, the *GRN* gene is also highly expressed in microglia.^51^ Loss of PGRN in *Grn*^−/−^ mice causes inflammation, astrocytosis, and microgliosis, which has been linked to synaptic loss and disease progression.^32,52^ In the *Grn*^−/−^ thalamic proteome, we find that many of the most dysregulated proteins are expressed by microglia, including GPNMB, CD68, and P2RY12 (Figure 2A). To examine this in more detail, we constructed a heatmap containing markers of microglial homeostasis, function, and activation in the proteome of *Grn*^+/+^ and *Grn*^−/−^ injected cohorts (Figure 4A).^53,54^ We found multiple markers of microglial activation, including CD45 and CD68, were upregulated in the GFP-*Grn*^−/−^ thalamus (Figures 4B and 4D). In addition, P2RY12, a marker of microglia homeostasis was downregulated in GFP-*Grn*^−/−^ mice (Figure 4C). Based on proteomics analyses, the elevation of CD45 was reduced by the expression of hGRN2, hGRN4, and hPGRN, while depressed P2RY12 levels were increased by hGRN4 and hPGRN (Figures 4B and 4C).

To expand our investigation to additional brain regions and validate proteomics, we examined levels of CD68, a type I transmembrane glycoprotein commonly used as a microglial activation marker^55^ and recently identified as a binding partner of PGRN,^56^ using IHC and immunoblotting in additional cohorts of mice. Immunohistochemical staining for CD68 revealed robust and significant increases in CD68 in the cortex, hippocampus, and thalamus of GFP-*Grn*^−/−^ mice (Figures 4D and 4E). Expression of hGRN4 in *Grn*^−/−^ mice lowered CD68 reactivity in all regions, while expression of hGRN2 significantly reduced levels in the thalamus and cortex but not the hippocampus (Figure 4E). Immunoblot of tissue lysates verified that CD68 levels were increased in the cortex and thalamus of GFP-*Grn*^−/−^ mice compared to GFP-*Grn*^+/+^ mice but below detection in the hippocampus (Figure 4F). Similar to immunohistochemical analysis, rAAV expression of both hGRN2 and hGRN4 corrected elevated CD68 levels relative to GFP-*Grn*^−/−^ in the cortex (Figure 4G) and thalamus (Figure 4H).

Finally, we asked if expression of GRNs corrected levels of GPNMB, the most elevated protein in the *Grn*^−/−^ brain thalamic proteome (Figure 2A), which was decreased by the expression of hGRN2 and hGRN4 in the thalamic proteomics (Figure 2E). GPNMB is a type 1 transmembrane glycoprotein that we discovered is highly upregulated by PGRN-deficient microglia.^42^ The function of GPNMB in microglia is unknown; however, GPNMB upregulation has been observed in activated damage-associated microglia^57^ and is functionally linked to lysosomal stress and lipid accumulation.^58,59^ We could not detect GPNMB via immunoblot at this age; therefore, we quantified murine GPNMB levels in thalamic tissue lysates using a validated ELISA.^42^ Using this approach, we found that expression of either hGRN2 or hGRN4 corrected elevated GPNMB levels to the same extent as hPGRN in *Grn*^−/−^ mice (Figure 4I). In sum, proteomics and multiple orthogonal biochemical measurements in separate cohorts of mice reveal that expression of hGRN2, hGRN4, and hPGRN, especially in the thalamus, decrease microglial activation in *Grn*^−/−^ mice.

### Lysosomal lipid dysregulation is rescued by a single GRN

The role of GRNs in the lysosome is not fully understood. Previous studies identified lipid dysregulation in PGRN-deficient animal models and FTD*-GRN* patient samples, suggesting that lysosomal metabolism of lipids is impaired.^12,60–62^ In particular, *Grn*^−/−^ mice display decreased levels of bis(monoacylglycerol) phosphate (BMP), an atypical endo-lysosomal lipid, and increased levels of glucosylsphingosine (GlcSph), a substrate of glucocerebrosidase (GCase), which can be corrected by the administration of exogenous full-length hPGRN.^63^ Additionally, ganglioside levels increase in brain tissues from *Grn*^−/−^ mice and patients with FTD-*GRN*,^64^ which may accumulate as secondary storage material, a phenomenon observed in many LSDs.^65,66^ To validate these observations and determine whether the expression of a single GRN or full-length hPGRN can correct them, we performed lipidomics and metabolomics analyses on the cortex of GFP-*Grn*^+/+^, GFP-*Grn*^−/−^, hPGRN-*Grn*^−/−^, hGRN2-*Grn*^−/−^, and hGrn4-*Grn*^−/−^ mice (see Tables S3, S4, S5, S6, and S7).

**Figure 5.**
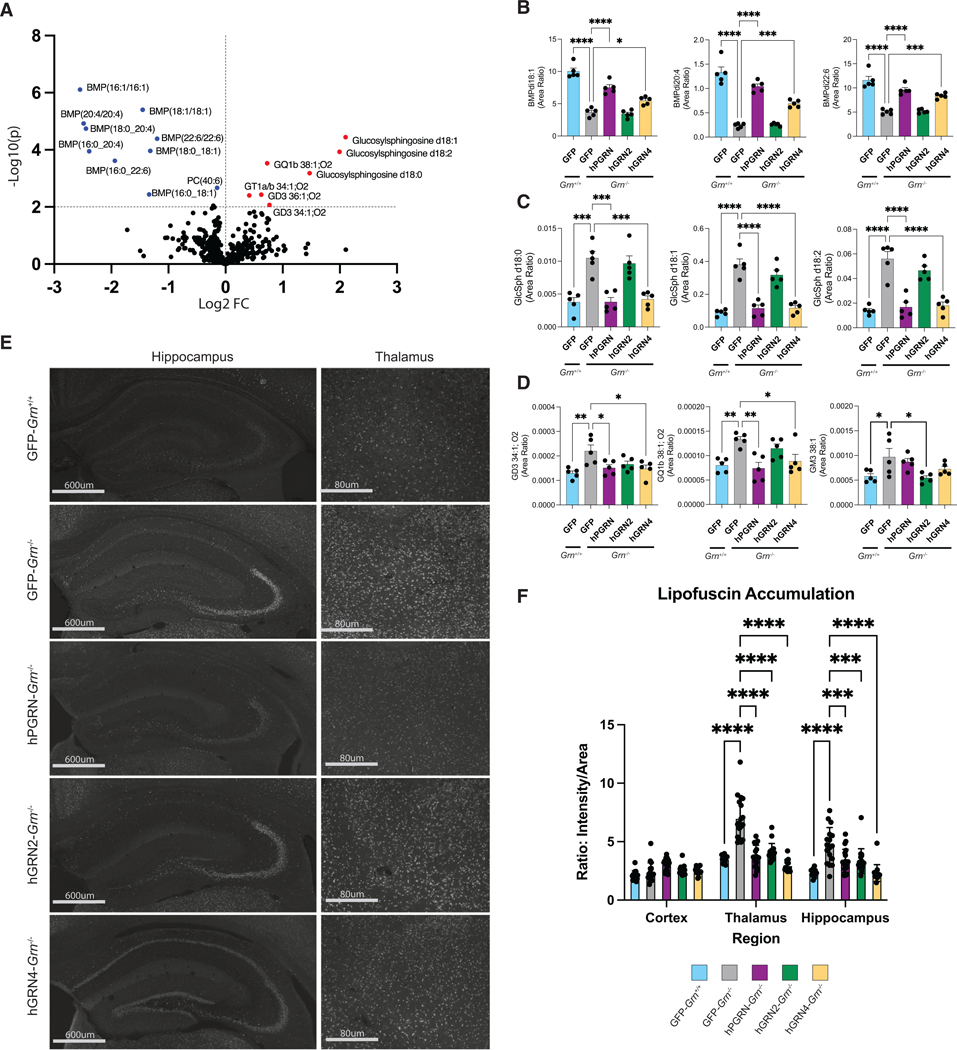
GRNs correct dysregulated lipids and prevent pathological accumulation of autofluorescent lipofuscin in Grn^−/−^ mouse brains (A) Volcano plot of lipids or metabolites upregulated (red) or downregulated (blue) in GFP-*Grn*^−/−^ mouse brain cortex compared to GFP-*Grn*^+/+^. (B) Quantification of differentially abundant BMP species. (C) Quantification of differentially abundant glycosphingosine species. (D) Quantification of differentially abundant gangliosides species. (E) Representative lipofuscin autofluorescence from hippocampal and thalamic regions of coronal sections from all injection groups presented in grayscale. (F) Quantification of fluorescent lipofuscin signal from cortex, hippocampus, and thalamus across all injected groups. Data are presented as mean ± SD. *n* = 5–7 mice/group. *p* values were determined by one-way or two-way (F) ANOVA with Tukey’s post hoc analysis. *p < 0.05, **p < 0.01, ***p < 0.001, and ****p < 0.0001.

We saw a decrease in the levels of BMP species and an increase in the levels of GlcSph species in GFP-*Grn*^−/−^ mice compared with GFP-*Grn*^+/+^ mice (Figure 5A). In addition, we observed smaller but significant increases of several gangliosides in the GFP-*Grn*^−/−^ mouse brain (Figure 5A), similar to previous reports.^64^ AAV-mediated expression of hGRN4 and hPGRN, but not hGRN2, in the *Grn*^−/−^ mouse brain significantly increased all measured BMP species back toward levels found in GFP-*Grn*^+/+^ mice (Figure 5B). Similarly, we found that both hGRN4 and hPGRN corrected the elevation of GlcSph (Figure 5C) and gangliosides (Figure 5D) in the GFP-*Grn*^−/−^ mouse brain. hGRN2 showed a slight but non-significant reduction in GlcSph and ganglioside levels as well. It is unclear why hGRN2 did not rescue BMP and gangliosides to the same extent as hGRN4 and hPGRN. This could be due to lower expression of hGRN2 (Figure S5C) or that different GRNs may have specific functions in lysosomal lipid metabolism. Overall, these results show that a single GRN can correct multiple lysosomal lipids that are dysregulated in the *Grn*^−/−^ mouse brain to the same extent as full-length hPGRN. Further research is needed to understand the specific functions of different GRNs on lysosomal lipid metabolism.

### Lipofuscin accumulation in *Grn*^−/−^ brains is alleviated by expression of hGRNs

Autofluorescent lipofuscin is a marker of lysosome dysfunction and a neuropathologic feature of human FTD-*GRN* and *Grn*^−/−^ mouse brain tissue.^35,67,68^ We set out to evaluate the extent and anatomical location of lipofuscin neuropathology in our rAAV-injected *Grn*^−/−^ mouse brain cohorts. We imaged whole coronal sections using Cy3 excitation and emission filters to capture autofluorescence in GFP-*Grn*^+/+^, GFP-*Grn*^−/−^, hPGRN-*Grn*^−/−^, hGRN2*-Grn*^−/−^, and hGRN4-*Grn*^−/−^ mice. Fluorescent signal in the cortex, hippocampus, and thalamic regions of all AAV-injected mice was then quantified using CellProfiler (Figure 5E). We observed a robust increase in lipofuscin in GFP-*Grn*^−/−^ animals compared with GFP*-Grn*^+/+^ animals in the thalamus and hippocampal regions but not the cortex (Figures 5E and 5F). Next, we found that the expression of hGRN2, hGRN4, and hPGRN decreased lipofuscin accumulation in the thalamus and hippocampus compared to GFP-*Grn*^−/−^ mice (Figures 5E and 5F). These data agree with our proteomic and lipidomic analyses and provide additional evidence that individual GRNs can ameliorate widespread lysosomal dysregulation in *Grn*^−/−^ mice, including the accumulation of lipofuscin, which has been linked to neurotoxicity and neurodegeneration in LSDs.^35^

### GRNs localize to the lysosome in cells and mouse brain

We show that AAV-mediated delivery of a single GRN corrects a broad spectrum of pathological changes in the *Grn*^−/−^ mouse brain. Our working hypothesis posits that the localization of GRNs to the lysosome is necessary to mediate beneficial activity and correct pathological phenotypes. To provide insight into this potential mechanism, we asked whether GRNs localized to the lysosome *in vivo* or *in vitro* following viral transduction.

**Figure 6.**
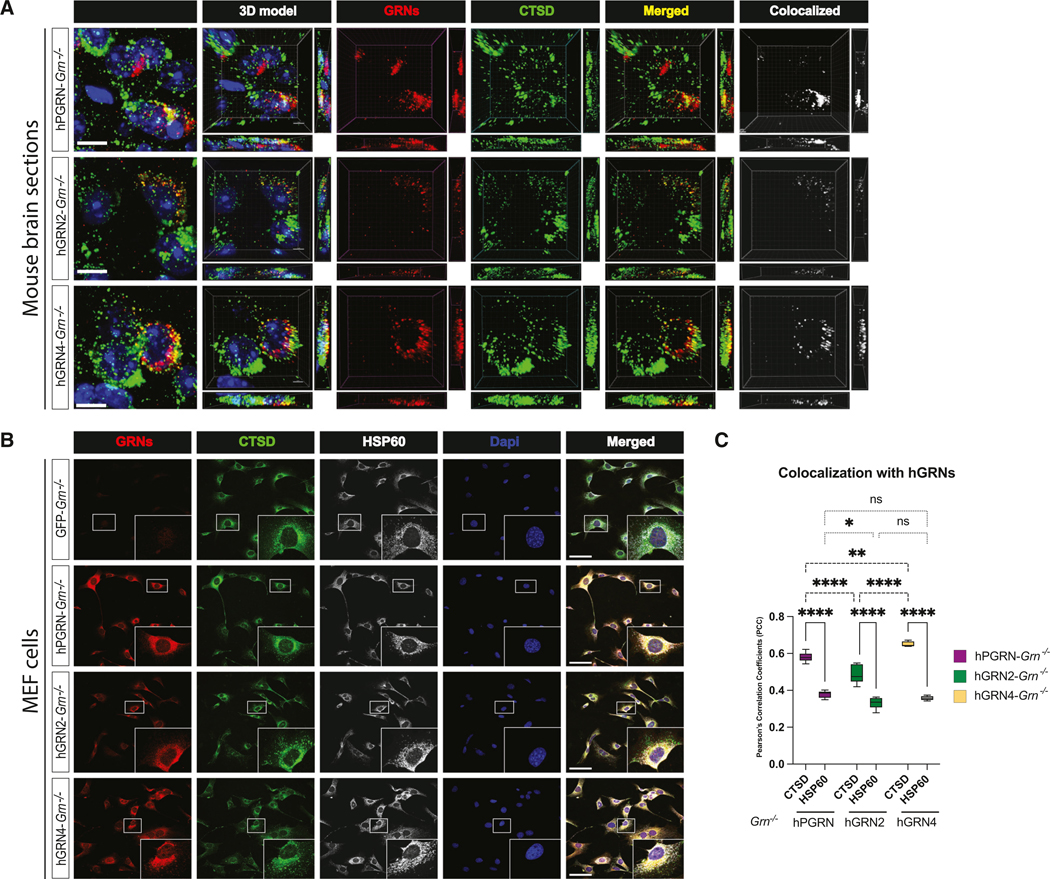
GRNs localize to the lysosome in mouse brain and cultured cells (A) Representative images of fluorescent immunohistochemistry of *Grn*^−/−^ mice injected with AAV-hPGRN, AAV-hGRN2, or AAV-hGRN4 stained for hGRNs (hPGRN, hGRN2, or hGRN4; red), lysosomal protein cathepsin D (CTSD; green), and nucleus (DAPI stain; blue). Images were analyzed with IMARIS software for voxel co-localization (white). Scale bar: 10 μm. (B) Representative images of fluorescent immunocytochemistry of MEF *Grn*^−/−^ TMEM192 3xHA cells expressing hPGRN, hGRN2, and hGRN4 stained for lysosomal protein CTSD (green), hGRNs (PGRN, GRN2, or GRN4; red), mitochondrial protein heat shock protein 60 (HSP60; gray), and nucleus (DAPI stain; blue). Scale bar: 10 μm. (C) Quantification of Pearson’s correlation coefficients (PCCs) between CTSD and hGRNs vs. HSP60 and hGRNs in MEF *Grn*^−/−^ TMEM192–3xHA cells expressing hPGRN, hGRN2, or hGRN4. Data are represented as mean ± SD. *n* = 5 area/group. *p* values were determined by two-way ANOVA with Tukey’s post hoc analysis. *p < 0.05, **p < 0.01, ***p < 0.001, and ****p < 0.0001.

We performed fluorescent IHC to determine if GRNs colocalize with cathepsin D (CTSD), a luminal lysosomal hydrolase, in *Grn*^−/−^ mouse brains injected with AAV encoding hPGRN, hGRN2, or hGRN4. We observed widespread punctate and vesicular staining of CTSD indicative of lysosomal localization.^69,70^ In cells positive for CTSD (green) and hPGRN, hGRN2, or hGRN4 (red), we observed frequent overlapping signals (yellow) suggestive of co-localization (Figure 6A). To assess the co-localization of hGRN and CTSD signals, we utilized IMARIS software to build three-dimensional (3D) models of transduced cells, then detected co-localizing voxels (white). In *Grn*^−/−^ mice brains injected with AAV-hPGRN, AAV-hGRN2, and AAV-hGRN4, we observed co-localization of all three proteins with CTSD, indicating that GRNs localize to the lysosome *in vivo* (Figure 6A).

**Figure 7.**
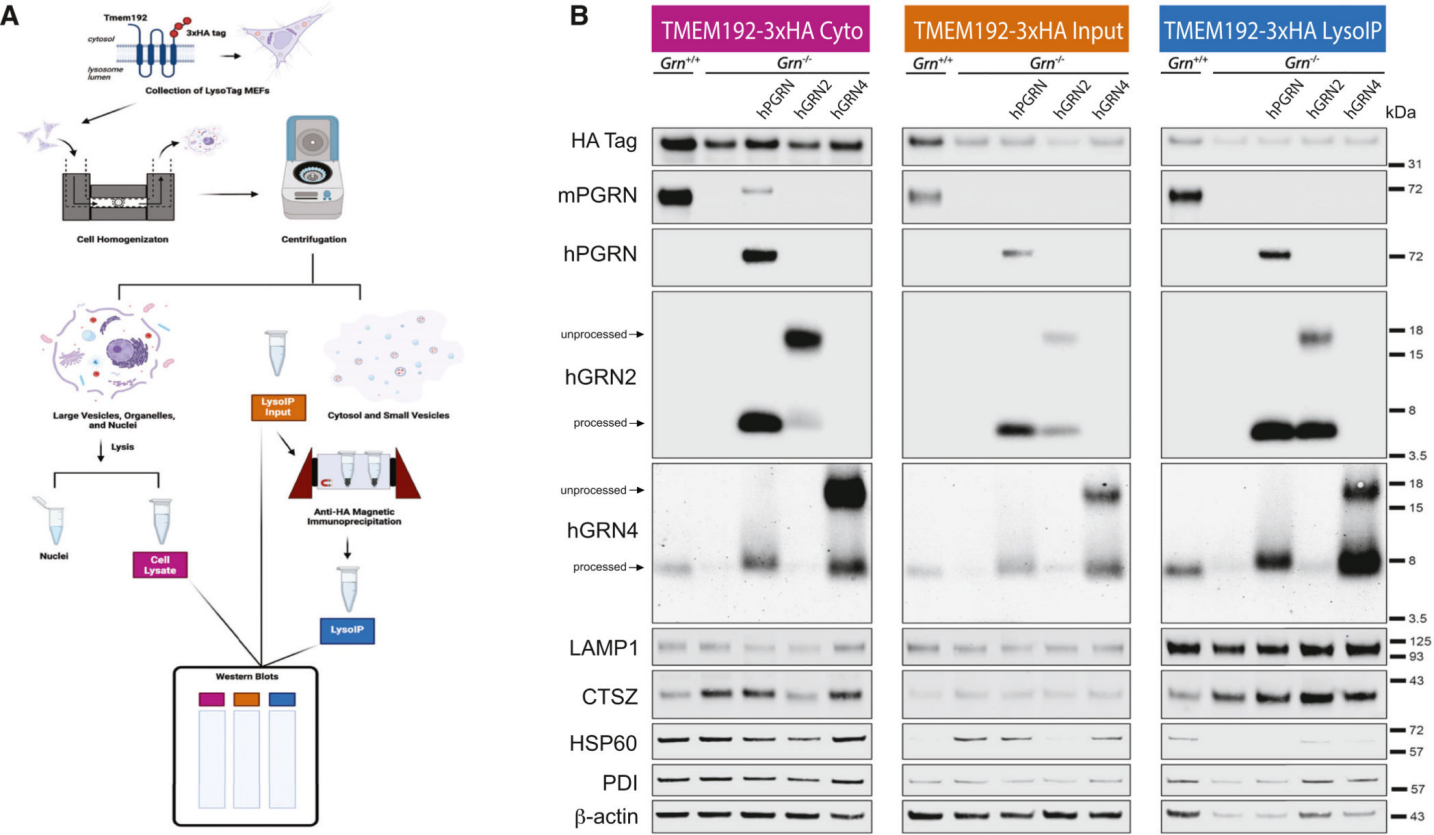
GRNs are enriched in the lysosome following lyso-IP (A) Lysosome immunoprecipitation (lyso-IP) workflow using MEF *Grn*^−/−^ cells co-expressing TMEM192–3xHA and hPGRN, hGRN2, or hGRN4. (B) Immunoblots of cell lysate (cyto), input, and lyso-IP fractions isolated from MEF *Grn*^−/−^ TMEM192–3xHA cells expressing hPGRN, hGRN2, and hGRN4 probed for lyso-tag (HA), mouse PGRN, hPGRN, hGRN2, hGRN4, lysosome (LAMP1 and CTSZ), mitochondria (HSP60), endoplasmic reticulum (PDI), and cytoskeleton (β-actin).

We then asked if we could visualize the co-localization of GRNs with lysosomal markers in cells (Figure 6) or detect GRNs in isolated lysosomes (Figure 7). We generated mouse embryonic fibroblast (MEF) *Grn*^−/−^ cells expressing TMEM192–3xHA, which enables lysosome immunoprecipitation (lyso-IP).^71^ MEF *Grn*^−/−^ TMEM192–3xHA lines were transduced with lentivirus to generate cells expressing hPGRN, hGRN2, or hGRN4, and then we performed triple-color fluorescent immunocytochemistry to determine if GRNs (red) co-localized with lysosomes (CTSD; green) or mitochondria (HSP60; gray) (Figure 6B). We observed robust overlapping signals (yellow), indicating the co-localization of CTSD with hPGRN, hGRN2, and hGRN4 (Figure 6B). In contrast, the immunofluorescent signal for mitochondria (HSP60) did not overlap with hPGRN, hGRN2, or hGRN4. We quantified the degree of co-localization by determining the Pearson’s correlation coefficient (PCC) and observed a significant co-localization of GRNs with CTSD compared to HSP60 (Figure 6C). Of note, the PCCs for CTSD were higher for hPGRN- and hGRN4- compared to that of hGRN2-transduced cells, suggesting there may be differences in lysosome trafficking. Higher-magnification images of these cells allow for the visualization of CSTD-positive lysosomes that co-localize with PGRN, hGRN2, and hGRN4 (Figure S7). These data show that the immunofluorescent signal for GRNs is predominantly localized in lysosomes in MEF *Grn*^−/−^ cells expressing hPGRN, hGRN2, or hGRN4.

To generate an orthogonal measure of lysosome localization, we performed lyso-IP on MEF *Grn*^−/−^ TMEM192–3xHA cell lines expressing hPGRN, hGRN2, or hGRN4 using anti-HA magnetic beads (Figure 7A).^71^ Lysosome isolation was verified using immunoblot to confirm the enrichment of LAMP1 and CTSZ and the depletion of other organelles, including mitochondria (HSP60), cytoskeleton (β-actin), and endoplasmic reticulum (PDI) (Figure 7B).^71^ As expected, hPGRN is cleaved into GRN2 and GRN4, and both GRNs are enriched in the lysosome by lyso-IP.^26^ In MEF *Grn*^−/−^ TMEM192–3xHA cells expressing hGRN2 or hGRN4, we observe the unprocessed (~15 kDa) precursor in the cell lysate and input, while the cleaved hGRN2 fragment (~6 kDa) or hGRN4 fragment (~8 kDa) is enriched in the lyso-IP sample. Thus, using lyso-IP of MEFs, we find that cleaved GRN2 and GRN4, whether generated from viral expression of hPGRN, hGRN2, or hGRN4, are enriched in the lysosome. In conclusion, both fluorescent immunostaining and lyso-IP reveal that hGRNs are present in the lysosome, providing evidence that the amelioration of disease-associated phenotypes in *Grn*^−/−^ mice is mediated by the lysosomal function of GRNs.

## DISCUSSION

Although it is well established that pathogenic *GRN* mutations decrease PGRN levels and cause neurodegeneration, the precise function of PGRN is still unclear. In general, full-length PGRN has been proposed to be neurotrophic and anti-inflammatory and function by activating extracellular signaling receptors.^4,19^ However, this hypothesis does not explain why complete PGRN deficiency causes an LSD. Based on our discovery that PGRN is processed into individual GRNs in the lysosome,^72^ we tested the idea that the lysosomal GRNs themselves are active. We find that the delivery of an individual GRN (~6 kDa) using rAAV is equally efficacious as hPGRN (~88 kDa) correcting a variety of neuropathologies in *Grn*^−/−^ mice. This fills a critical gap in our knowledge of PGRN biology, strongly supporting the idea that individual GRNs are the bioactive components of PGRN.

This is an important conceptual advance because, prior to this study, GRNs had been proposed to have the opposite activity of PGRN by causing inflammation,^17^ neurotoxicity,^24^ or lysosomal defects.^25^ In contrast to these competing hypotheses, we find that the expression of two different GRNs in the *Grn*^−/−^ mouse brain was neuroprotective and reduced markers of neuroinflammation and glial activation. Additional support for our data comes from a study of PTV:PGRN, a brain-penetrant form of hPGRN, that was efficacious weeks after dosing *Grn*^−/−^ mice, when PGRN has been degraded, suggesting that stable, lysosomal GRNs mediate prolonged efficacy.^63^ These findings suggest that GRNs have a central role in regulating lysosomal function and lysosomal lipid metabolism and may hold therapeutic potential for neurodegenerative diseases with lysosome dysfunction.^73–75^

Intriguingly, we find that while neurons are the predominant cell expressing GRNs, we see a rescue of microglial phenotypes (Figure 4), suggesting that neuronal GRNs in *Grn*^−/−^ mice may have beneficial cell-autonomous and non-cell-autonomous effects. Because the hGRN2 and hGRN4 constructs we employed are secreted (Figure S2B), it is likely they can be taken up by neighboring brain cells to correct dysfunctional lysosomes and gliosis. While we did not see widespread co-localization of hGRNs with microglia (Figure 1F), we did observe rare co-localization (Figure S4B) suggesting some hGRNs may be endocytosed by microglia. In agreement with our data, another study found that AAV-neuronal expression of hPGRN in *Grn*^−/−^ mice rescued gliosis.^68,76^ Further experiments with more sensitive techniques are necessary to dissect how neuronal expression of GRNs is beneficial to microglia in *Grn*^−/−^ mice.

Our findings have important implications for therapeutic development to treat FTD-*GRN,* AD, and PD. Multiple strategies to increase PGRN in the CNS are being pursued for clinical development, including protein replacement,^12^ gene therapy,^45^ and small molecules.^77^ One approach aims to increase PGRN by depleting sortilin (SORT1), a PGRN lysosomal trafficking receptor, with an antibody (AL001) that has advanced to a phase 3 clinical trial (ClinicalTrials.gov: NCT04374136).^11^ Antibodies targeting SORT1 increase circulating PGRN in mice^78^ but decrease lysosomal GRNs in induced pluripotent stem cell (iPSC)-derived neurons.^79^ Our data raise concerns about this approach because anti-SORT1 antibodies likely raise extracellular PGRN by reducing lysosomal trafficking and decreasing GRN production, which we find prevent lysosome dysfunction caused by PGRN deficiency. At a minimum, our data raise a cautionary note that both PGRN and intra-lysosomal GRN levels should be measured when evaluating pre-clinical therapeutic approaches to treat PGRN deficiency in humans.

Although the precise mechanism through which GRNs are beneficial remains an active area of investigation, we hypothesize that GRNs function inside the lysosome to prevent lipid accumulation, leading to the amelioration of FTD-associated phenotypes. In support of this hypothesis, we show that both hGRN2 and hGRN4 co-localize with the lysosomal protein CSTD in *Grn*^−/−^ mice and MEF cells. Further, we find that cleaved GRNs are enriched in the lysosomes following lyso-IP of MEF *Grn*^−/−^ cells expressing hGRN2, hGRN4, or hPGRN. These findings demonstrate that AAV-hGRNs expressed in *Grn*^−/−^ mice reach the lysosome and support the hypothesis that the lysosomal function of GRNs is responsible for neuroprotection and rescue of lysosome dysfunction and inflammation.

Key questions remain, including whether all GRNs can rescue pathologic phenotypes caused by PGRN deficiency. Further, the molecular function of GRNs inside the lysosome is unsolved, and it is unclear whether each GRN has a unique or overlapping activity. It is feasible that GRNs could contribute to lysosomal function through the stabilization of BMP, which has been suggested as a role of PGRN previously,^63^ or, similar to the role of saposins, as a co-factor or chaperone in BMP synthesis. Because BMP is a unique and critical lipid for lysosome function, which promotes hydrolase activity,^80^ rescue of depressed BMP levels could drive the beneficial effect observed with the expression of GRNs. Further experimentation is necessary to test these possibilities and see if all GRNs participate in the same molecular pathways.

From a therapeutic perspective, the small size of GRNs may be advantageous for treating FTD-*GRN* by crossing the bloodbrain barrier more readily than PGRN. Furthermore, therapies that aim to raise PGRN levels need to consider the impact on GRN levels throughout the CNS. Finally, our data suggest that understanding the function of GRNs inside the endosomal-lysosomal pathway is necessary to decipher how GRNs mediate lysosomal homeostasis and prevent neurodegeneration in FTD-*GRN* and related disorders.

### Limitations of the study

In this study, we tested the efficacy of two out of the seven GRNs in *Grn*^−/−^ mice. Despite this limitation, we found that hGRN2 and hGRN4 equally corrected major markers of lysosome dysfunction, microglial activation, and lipofuscin in *Grn*^−/−^ mouse brains. However, in two cases, correction of BMP and GlcSph lipids, hGRN2 was not as efficacious as hGRN4 or hPGRN. Proteomics quantification suggests that this may be due to a 2.5-fold higher expression of hGRN4 compared to hGRN2, which may have limited the efficacy of hGRN2. Alternatively, hGRN2 and hGRN4 may have distinct lysosomal functions, half-lives,^81^ or lysosomal trafficking efficiency. Although lysosomal receptors for PGRN are known,^19,82,83^ it is unclear how hGRN2 or hGRN4 traffic to the lysosome and is a focus of future studies. Despite these unanswered questions, the shared ability of hGRN2 and hGRN4 to rescue neuropathology in *Grn*^−/−^ mice strongly supports further investigation of the bioactivity of all GRNs *in vivo.*

### RESOURCE AVAILABILITY

#### Lead contact

Requests for reagents will be fulfilled by the lead contact, Thomas Kukar (tkukar@emory.edu).

#### Materials availability

Plasmids generated in this study are deposited at Addgene. Cell lines and antibodies are available upon request.

#### Data and code availability

- Proteomic data are publicly available and deposited to the ProteomeXchange Consortium via the PRIDE repository (http://www.ebi.ac.uk/pride/archive/projects/PXD041095).
- Metabolomic and lipidomic data are publicly available and deposited at Dataverse (https://doi.org/10.15139/S3/7Z2SVL).
- No original code was produced, and all scripts and packages used to analyze data have been included in the key resources table.

## STAR★METHODS

### EXPERIMENTAL MODEL AND SUBJECT DETAILS

#### Mouse model and neonatal rAAV injections

The *Grn*^−/−^ mice used in this study were purchased from the Jackson Laboratory (B6(Cg)-Grntm1.1Aidi/, IMSR Cat# JAX:013175, RRID: IMSR_JAX:013175) and generated as previously described. Mice were bred and housed in the Department of Animal Resources at Emory University and all work was approved by the Institutional Animal Care and Use Committee (IACUC) and performed in accordance with the Guide for the Care and Use of Laboratory Animals of the National Institutes of Health. Postnatal day 0 (P0) mouse pups (*GRN*^+/+^ or *GRN*^−/−^) were injected with rAAV vectors.^41^ Briefly, P0 pups were cryoanesthetized on ice for 5 min in a nest protected by aluminum foil. One microliter of rAAV was injected intracerebroventricular (ICV) into both hemispheres using a 10 mL Hamilton syringe with a 30-gauge needle. The pups were then placed on a heating pad with their original nesting material for 3–5 min and returned to their mother for further recovery. Male and female mice were injected in a randomized manner. Treatment group was determined by litter, as one litter received a single virus type on a rotating basis. Between 2 and 3 litters were injected with each construct to achieve cohort size. Data from 32 females and 26 males are included in this study.

#### Cell culture

HeLa *GRN*^−/−^ cells were a gift from Dr. Shawn Ferguson (Yale University) and generated using CRISPR as described.^78^ The progenitors of modern HeLa cells were collected at John’s Hopkins from cervical cancer patient Henrietta Lacks in 1951 an African American woman living near Baltimore. The cells were collected without her or her family’s knowledge or consent, we encourage all who use these cell lines to acknowledge and consider the history of these cells.^92^ HeLa wild-type or *GRN*^−/−^ cells were cultured in DMEM medium plus 10% fetal bovine serum (FBS) and 1% Pen/Strep and maintained at 37°C with 5% CO2. 24 h before collection DMEM media was replaced with Opti-MEM media (Gibco).

HEK293T cells used to generate lentiviral constructs were acquired from ATCC (RRID: CVCL_0063). This cell line originated from female embryonic kidney cells.^93^ Cells were cultured in DMEM medium plus 10% fetal bovine serum (FBS) and 1% Pen/Strep and maintained at 37°C with 5% CO2.

MEF *Grn*^+/+^ and *Grn*^−/−^ cells (parental lines), a gift from Dr. Robert Farese (Harvard University), were cultured in DMEM medium, supplemented with 10% heat-inactivated fetal bovine serum (hi-FBS) at 37°C and 5% CO_2_ environment. Cells were cultured for two passages after thaw and prior to lentiviral transduction to generate stable line for lysosomal immunoprecipitation (Lyso-IP). Cell count was estimated using a TC20 Automated Cell Counter (Bio-Rad).

### METHOD DETAILS

#### Production of recombinant adeno-associated virus

Four purified recombinant adeno-associated virus vectors (rAAVs) for injection were produced by plasmid transfection with helper plasmids in HEK293T cells. Briefly, the coding sequence of twin-Strep-GFP (GFP), twin-Strep-V5 human progranulin (hPGRN), twin-Strep-FLAG-granulin-2 with linker region 3 (hGRN2) and twin-Strep-FLAG-granulin-4 with linker region 5 (hGRN4) were subcloned from a pcDNA3.1 expression plasmid into pAAV. hPGRN, hGRN2, and hGRN4 all contain the native hPGRN signal peptide at the N terminus. The AAV vectors express hPGRN, hGRN2, hGRN4, or GFP under the control of the cytomegalovirus enhancer/chicken β-actin promoter, a woodchuck post-transcriptional regulatory element, and the bovine growth hormone, poly(A), and were generated by plasmid transfection with helper plasmids in HEK293T cells. Forty-eight hours after transfection, the cells were harvested and lysed in the presence of 0.5% sodium deoxycholate and 50 U/ml Benzonase (Sigma, St. Louis, MO) by freeze thawing, and the virus was isolated using a discontinuous iodixanol gradient and affinity purified on a HiTrap HQ column (Amersham Biosciences, Arlington Heights, IL). The genomic titer of each virus was determined by quantitative PCR.

#### Collection of brain tissue

Mice were sacrificed after 12 months and brains were processed in two downstream pathways. Brains from half of the individuals from each cohort were immediately dissected from the skull and frozen at −80C. Whole brains were later thawed on ice and cortical, hippocampal, and thalamic sections were bulk dissected from the brain and frozen immediately at −80C. Remaining animals were transcardially perfused using ice-cold PBS then fixed in methanol-free 4% PFA before dissecting all brains and storing in 4% PFA for 24 h before transferring samples to 30% sucrose, which was replaced at 24 and 48 h. The final storage solution was 30% sucrose and 1% sodium azide. Fixed tissue was stored at 4°C until it was prepared for sectioning. Brain sectioning was performed using a freezing microtome set to 40 μm. Brains were frozen in ground dry ice, then mounted with 30% sucrose onto the pre-frozen sectioning stage, where serial sections were collected from the entire brain and stored in 30% sucrose, 30% ethylene glycol and 1% sodium azide. Frozen hippocampal and cortical brain samples from 12-month-old *Grn*^+/+^ (*n* = 27) and *Grn*^−/−^ (*n* = 44) mice were allocated for further processing.

#### Thalamic proteomics sample preparation

Each tissue sample was homogenized in 300 μL of 8 M urea/100 mM NaHPO_4_, pH 8.5 with HALT protease and phosphatase inhibitor cocktail (Pierce) using a Bullet Blender (Next Advance) according to manufacturer protocols. Briefly, tissue lysis was transferred to a 1.5 mL Rino tube (Next Advance) with 350 mg stainless steel beads (0.9–2 mm in diameter) and blended for 5-min intervals, two times, at 4°C. Protein supernatants were sonicated (Sonic Dismembrator, Fisher Scientific) three times for 5 s, with 15 s intervals of rest, at 30% amplitude to disrupt nucleic acids, in 1.5 mL Eppendorf tubes. Protein concentration was determined by BCA method, and aliquots were frozen at −80°C. Protein homogenates (200 μg) were treated with 1 mM dithiothreitol (DTT) at 25°C for 30 min, followed by 5 mM iodoacetimide (IAA) at 25°C for 30 min in the dark. Proteins were digested with 1:25 (w/w) lysyl endopeptidase (Wako) at 25°C for overnight followed by another overnight digestion with 1:25 (w/w) trypsin (Pierce) at 25°C after dilution with 50 mM NH_4_HCO_3_ to a final concentration of 1 M urea. The resulting peptides were desalted on a Sep-Pak C18 column (Waters) and dried under vacuum. All samples were across 2 batches and labeled with an 18-plex Tandem Mass Tag (TMTPro) kit (ThermoFisher, Lot numbers: UK297033 and WI336758) according to manufacturer’s protocol. Each TMT batch was desalted with 60 mg HLB columns (Waters) and dried via speed vacuum (Labconco). Dried samples were re-suspended in high pH loading buffer (0.07% v/v NH_4_OH, 0.045% v/v FA, 2% v/v ACN) and loaded onto a Water’s BEH column (2.1 mm × 150 mm with 1.7 μm particles). A Vanquish UPLC system (ThermoFisher Scientific) was used to carry out the fractionation. Solvent A consisted of 0.0175% (v/v) NH_4_OH, 0.01125% (v/v) FA, and 2% (v/v) ACN; solvent B consisted of 0.0175% (v/v) NH_4_OH, 0.01125% (v/v) FA, and 90% (v/v) ACN. The sample elution was performed over a 25 min gradient with a flow rate of 0.6 mL/min with a gradient from 0 to 50% solvent B. A total of 192 individual equal volume fractions were collected across the gradient. Fractions were concatenated to 96 fractions and dried to completeness using vacuum centrifugation. Dried peptide fractions were resuspended in 20 μL of peptide loading buffer (0.1% formic acid, 0.03% trifluoroacetic acid, 1% acetonitrile). Peptide mixtures (2 μL) were separated on a self-packed C18 (Dr. Maisch) fused silica column (15 cm × 150 μm internal diameter) by a Dionex Ultimate rsLCnano and monitored on a Fusion Lumos mass spectrometer (ThermoFisher). Elution was performed over a 42 min gradient at a rate of 1250 nL/min with buffer B ranging from 1% to 99% (buffer A: 0.1% formic acid in water, buffer B: 0.1% formic in 80% acetonitrile). The mass spectrometer cycle was programmed to collect at the top speed for 3 s cycles. The MS scans (410–1600 m/z range, 400,000 AGC, 50 ms maximum ion time) were collected at a resolution of 60,000 at m/z 200 in profile mode. HCD MS/MS spectra (0.7 m/z isolation width, 35% collision energy, 125,000 AGC target, 86 ms maximum ion time) were collected in the Orbitrap at a resolution of 50000. Dynamic exclusion was set to exclude previous sequenced precursor ions for 20 s within a 10-ppm window. Precursor ions with +1 and +8 or higher charge states were excluded from sequencing.

#### Thalamic proteomics data processing

All raw files were analyzed using the Proteome Discoverer Suite (v.2.4.1.15, ThermoFisher). MS/MS spectra were searched against the UniProtKB mouse proteome database (downloaded in August 2020 with 91417 total sequences) supplemented with 4 variant sequences (twin-Strep-GFP, hGRN2, hGRN4, and hPGRN). The Sequest HT search engine was used to search the RAW files, with search parameters specified as follows: fully tryptic specificity, maximum of two missed cleavages, minimum peptide length of six, fixed modifications for TMTPro tags on lysine residues and peptide N-termini (+304.207 Da) and carbamidomethylation of cysteine residues (+57.02146 Da), variable modifications for oxidation of methionine residues (+15.99492 Da), serine, threonine and tyrosine phosphorylation (+79.966 Da) and deamidation of asparagine and glutamine (+0.984 Da), precursor mass tolerance of 10 ppm and a fragment mass tolerance of 0.05 Da. Percolator was used to filter peptide spectral matches and peptides to an FDR <1%. Following spectral assignment, peptides were assembled into proteins and were further filtered based on the combined probabilities of their constituent peptides to a final FDR of 1%. Peptides were grouped into proteins following strict parsimony principles.

#### Differential expression analysis

Differentially enriched or depleted proteins (*p* % 0.05) were identified by one-way ANOVA with post-hoc Tukey HSD test comparing five groups: GFP-*Grn*^+/+^, GFP-*Grn*^−/−^, PGRN-*Grn*^−/−^, GRN2-*Grn*^−/−^, GRN4-*Grn*^−/−^ mice. Differential expression of proteins was visualized with volcano plots generated using the ggplot2^94^ package in Microsoft R Open v3.4.2. Significantly differentially expressed proteins were determined by both having a *p* % 0.05 and a fold change difference of greater than log2(1.25) or less than − log2(1.20) (a minimum 1.2-fold change).

#### Proteomics analysis and visualization

Differential expression data from comparisons of GFP-Grn^+/+^ vs. GFP-*Grn*^−/−^, GFP-*Grn*^−/−^ vs. hPGRN-*Grn*^−/−^, GFP-*Grn*^−/−^ vs. hGRN2-*Grn*^−/−^, and GFP-*Grn*^−/−^ vs. hGRN4-*Grn*^−/−^ including adjusted *p* values, and abundance values were imported into Quickomics, an R-shiny powered proteomics analysis and visualization tool.^91^ GIS internal standards were removed from the dataset and Heatmaps were created filtering proteins from the GFP-*Grn*^+/+^ vs. GFP-*Grn*^−/−^ comparison with and adjusted *p*-value of <0.05 and a fold change value of at least 1.2 or 20%. Clustering was performed grouping proteins by the similarity across the sample ID using a k-means approach. Other visualizations created in Quickomics include 2-Way DEG plots and PCA visualizations. Additional PCA analysis was undertaken in R using the PCAtools package.^86^

#### Gene ontology (GO)

Genes IDs identified from proteins determined to be differentially abundant (adjusted *p*-value 0.05, FC 1.2) between GFP-*Grn*^+/+^, GFP-*Grn*^−/−^ mice were input into the Metascape Gene Ontology Analysis tool (https://metascape.org).^95^ Express Analysis was conducted, and the top 50 Ontology Terms were collected.

#### Lipidomics and metabolomics

##### Sample preparation for lipidomics and metabolomics analyses

During tissue collection, the cortex was dissected, weighed, and flash frozen. Each frozen cortex was pulverized into a homogeneous powder, and roughly 30 mg of each cortex powder sample was used to extract lipids. Methanol spiked with internal standards (see LCMS methods below) was added to each sample and homogenized with FastPrep-24 5G bead beating grinder and lysis system using Lysing Matrix D tubes with CoolPrep adapter (MP Biomedicals) for 40 s at a speed of 6 m/s. The methanol fraction was then isolated via centrifugation (20 min at 4°C, 14,000 x g), followed by transfer of supernatant to a 96 well plate. After a 1 h incubation at 20°C followed by an additional centrifugation (20 min, 4,000 x g at 4°C), methanol was transferred to glass vials for LCMS analysis.

##### Lipidomics analysis

Lipid analyses were performed by liquid chromatography on an ExionLC (Sciex) coupled with electrospray mass spectrometry TripleQuad 7500 (Sciex). For each analysis, 1 μL of the sample was injected on a Premier BEH C18 1.7 μm, 2.1 × 100 mm column (Waters) using a flow rate of 0.25 mL/min at 55°C. For positive ionization mode, mobile phase A consisted of 60/40 (v/v) acetonitrile/water with 10 mM ammonium formate +0.1% formic acid; mobile phase B consisted of 90/10 (v/v) isopropyl alcohol/acetonitrile with 10 mM ammonium formate +0.1% formic acid. For negative ionization mode, mobile phase A consisted of 60/40 (v/v) acetonitrile/water with 10 mM ammonium acetate; mobile phase B consisted of 90/10 (v/v) isopropyl alcohol/acetonitrile with 10 mM ammonium acetate. The gradient was programmed as follows: 0.0–8.0 min from 45% B to 99% B, 8.0–9.0 min at 99% B, 9.0–9.1 min to 45% B, and 9.1–10.0 min at 45% B. Electrospray ionization was performed in positive or negative ion mode. We applied the following settings: curtain gas at 40 psi (negative mode) and curtain gas at 40 psi (positive mode); collision gas was set at 9; ion spray voltage at 2000 V (positive mode) or −2000 V (negative mode); temperature at 250°C (positive mode) or 450°C (negative mode); ion source Gas 1 at 40 psi; ion source Gas 2 at 70 psi; entrance potential at 10 V (positive mode) or −10 V (negative mode); and collision cell exit potential at 15 V (positive mode) or −15 V (negative mode). Data acquisition was performed in multiple reaction monitoring mode (MRM) with the collision energy (CE) values reported in Tables S3 and S4. Area ratios of endogenous lipids and surrogate internal standards were quantified using SCIEX OS 3.1 (Sciex).

##### Metabolomics analysis

Metabolites analyses were performed by liquid chromatography on an ExionLC (Sciex) coupled with electrospray mass spectrometry TripleQuad 7500 (Sciex). For each analysis, 1 μL of the sample was injected on a Premier BEH amide 1.7 μm, 2.1 × 150 mm column (Waters) using a flow rate of 0.40 mL/min at 40°C. Mobile phase A consisted of water with 10 mM ammonium formate +0.1% formic acid. Mobile phase B consisted of acetonitrile with 0.1% formic acid. The gradient was programmed as follows: 0.0–1.0 min at 95% B; 1.0–7.0 min to 50% B; 7.0–7.1 min to 95% B; and 7.1–10.0 min at 95% B. Electrospray ionization was performed in positive ion mode. We applied the following settings: curtain gas at 40 psi; collision gas was set at 9; ion spray voltage at 1600 V; the temperature at 350°C; ion source Gas 1 at 30 psi; ion source Gas 2 at 50 psi; entrance potential at 10 V; and collision cell exit potential at 10 V. Data acquisition was performed in MRM mode with the CE values reported in Table S5. Area ratios of endogenous metabolites and surrogate internal standards were quantified using SCIEX OS 3.1 (Sciex).

##### Analysis of glucosyl- and galactosyl-sphingolipids

Glucosyl- and galactosyl-sphingolipids analyses were performed by liquid chromatography ExionLC coupled to electrospray mass spectrometry TQ7500. For each analysis, 1 μL of sample was injected on a HALO HILIC 2.0 μm, 3.0 × 150 mm column (Advanced Materials Technology) using a flow rate of 0.48 mL/min at 45°C. Mobile phase A consisted of 92.5/5/2.5 (v/v/vol) acetonitrile/isopropanol/water with 5 mM ammonium formate and 0.5% formic acid. Mobile phase B consisted of 92.5/5/2.5 (v/v/vol) acetonitrile/isopropanol/water with 5 mM ammonium formate and 0.5% formic acid. The gradient was programmed as follows: 0.0–2 min at 0% B, 2.1 min at 5% B, 4.5 min at 15% B, hold to 6.0 min at 15% B, up to 100% B at 6.1 min and hold to 7.0 min, drop back to 0% B at 7.1 min and hold to 8.5 min. Electrospray ionization was performed in positive ion mode. We applied the following settings: curtain gas at 40 psi; collision gas was set at 9 psi; ion spray voltage at 2250 V; temperature at 450°C; ion source Gas 1 at 40 psi; ion source Gas 2 at 70 psi; entrance potential at 10 V; and collision cell exit potential at 15 V. Area ratios of endogenous glucosyl- or galactosyl-sphingolipids and surrogate internal standards (Table S6) were quantified using SCIEX OS 3.1 (Sciex).

#### Generation of human granulin antibodies

Polyclonal antibodies that specifically recognize human granulin-2 or granulin-4 were generated by immunizing rabbits with recombinant antigen followed by affinity purification of sera. Briefly, synthetic genes were designed to encode a dual polyhistidine (His) and albumin binding protein (ABP) tag at the N terminus of hGRN2 or hGRN4. His-ABP-hGRN2 and His-ABP-hGRN4 were made recombinantly in *E. coli* Shuffle T7 cells (NEB). Proteins were sequentially purified over Ni^2+^-columns (cOmplete His-tagged resin; Millipore – Sigma) then a human serum albumin column (ProteinMods) following the manufacturer’s protocols under native condition. Purified His-ABP-hGRN2 and His-ABP-hGRN4 were used to immunize rabbits (*n* = 2) with complete Freund’s adjuvant by Pocono Rabbit Farm and Laboratory following their 70-day antibody production protocol. Rabbit sera that were immunopositive for hGRN2 or hGRN4 by ELISA and immunoblot were then purified over a protein A column to isolate IgG followed by a column (AminoLink; ThermoFisher Scientific) that had been pre-coupled with twin-strep-tagged hGRN2 or hGRN4 made in Expi293F cells (Thermo-Fisher Scientific) as previously described.^26^ Anti-hGRN2 or anti-hGRN4 rabbit antibodies were eluted with gentle Ag/Ab elution buffer, pH 6.6 (Thermo-Fisher Scientific). Elutions were pooled, dialyzed into PBS, then concentrated in a 50 kDa Amicon 4 mL ultra-centrifugal Filter (Millipore - Sigma).

#### Immunohistochemistry

Paraformaldehyde fixed coronal tissue sections from each group of rAAV-injected mice were stained with multiple antibodies including, StrepTagII C23.21 antibody, lysosomal proteins (CathZ, Gal3), and microglial protein CD68 using previously published procedures.^39,42^ The full list of antibodies is listed in the key resources table. For this procedure, 40 μm coronal brain sections were processed using a free-floating method. For StrepTagII and CD68 antibodies, antigen retrieval with Citrate buffer pH 6.0 at 80° (30 min) was performed for epitope retrieval. Sections were rinsed three times in phosphate-buffered saline containing 0.3% Triton X-100 (PBST) (0.1M Phosphate buffer, pH 7.4, 0.137 M NaCl, 0.3% Triton X-100) and reacted in PBST containing 1% hydrogen peroxide (30 min) to remove endogenous peroxidase activity, rinsed three times in PBST, blocked with 2.5% normal horse serum, and then incubated in optimal dilutions of antibody overnight with shaking at room temperature (RT). Sections were then rinsed three times, incubated in biotinylated anti-species immunoglobulin (Vector Laboratories) at 1:1000 for 1 h at room temperature, rinsed three times and then incubated with avidin-biotin-peroxidase complex (ABC) for an hour (Vector Laboratories). Localization of bound antibody was visualized using avidin-biotin horseradish peroxidase (HRP) enzyme complex histochemistry and nickel ammonium sulfate-enhanced diaminobenzidine-HCl (100 μg/mL) (TCI Chemicals, Tokyo, Japan) as a substrate to produce a dark purple reaction product. Sections were then mounted on microscope slides, dehydrated, and coverslipped with Pertex mounting medium.

For detection of the twin-Strep tag on constructs, the StrepTagII C23.21 antibody was visualized using the Mouse on Mouse ImmPress HRP Polymer kit (Vector Laboratories) according to the manufacturer’s protocol. Tissue slides were imaged with a slide scanner (Leica Aperio AT2) and the IHC signal of cortical, thalamic, and hippocampal regions were cropped from a whole coronal section image. Brain regions of interest analyzed using an automated pipeline created using CellProfiler (www.cellprofiler.org)^85^ for quantification.

#### Fluorescent immunohistochemistry

Double-color fluorescent immunohistochemistry was carried out to verify cellular co-localization of PGRN, GRN2, and GRN4-expressing cells in hGRNs-*Grn*^−/−^ mice with cellular antigenic markers such as neuronal marker (Map2), microglial marker (Iba-1), astrocyte marker (Gfap), and a lysosomal marker (CTSD). Tissue sections were rinsed three times in PBST, blocked with 2.5% normal donkey serum for 30 min, and followed by incubation with optimal dilutions of antibodies at 4^◦^ overnight with shaking. After three washes (10 min each) in PBST, sections were incubated with optimal concentrations of fluorescent-labeled secondary antibodies and DAPI (1ug/ml) mixture. Bound primary antibodies were detected with Alexa Fluor 488 donkey anti-chicken or anti-mouse IgG, Alexa Fluor 568-donkey anti-goat IgG, and Cy5-donkey anti-rabbit IgG. After three washes, sections were mounted, coverslipped with Immu-mount fluorescent mounting media (Thermo Fisher) and imaged using Lecia DMi 8 or Lecia STELLARIS SP8 microscope with a DFC9000 GT camera and system software (LAS X Life Science microscope software).

#### IMARIS colocalization

Three-dimensional visualization and analysis of colocalization between lysosomal marker, CTSD and granulins in hPGRN, hGRN2, and hGRN4 injected mouse brain tissue sections, we employed IMARIS (v.10.0, Bitplane). After acquiring images with a Leica SP8 Stellaris, the datasets were imported into IMARIS for volumetric reconstruction. Thresholding was set using fluorescent secondary antibody only stained sections to minimize background noise and optimize the accuracy of colocalization detection. We used the software’s ‘Coloc’ function to visualize colocalization within the reconstructed 3D models. A Colocalization channel was built, assigned to white color, and exported.

#### HeLa lysis and media collection

Cells were suspended in MES buffer (50 mM MES pH 6.5, 1% Triton, 150 μM NaCl, 1XHALT PPI) 5 μL for every 1 mg cell pellet. Cells were then lysed on ice for 10 min briefly vortexing every 3 min. Lysates were then spun at 600xg for 10 min and supernatant was collected. Conditioned media was collected from culture dish and spun at 500xg for 10 min to remove any cell debris.

#### Lentiviruses

pLJC5-Tmem192–3xHA (cat. 102930), pCMV-VSV-G (cat. 8454) and pCMV-dR8.2 dvpr (cat. 8455) were acquired from Addgene as bacterial stabs. Plasmids were propagated in TOP10 competent *E. coli* (Invitrogen), plasmid DNA was extracted and purified with a ZymoPURE II Plasmid Maxiprep kit (Zymo Research). TMEM192_3xHA lentiviruses were generated as previously described.^71^ Briefly, 500,000 HEK293T cells per well were seeded into 6-well plates in DMEM/10% hi-FBS. Cells were transfected the next day with the pLJC5-Tmem192– 3xHA, VSV-G and CMV- ΔVPR with TransIT-LT1 Transfection reagent (Mirus Bio). After an 18-h incubation period, growth medium was changed to DMEM/30% hi-FBS. 48 h later, the medium was collected, centrifugated at 1,000 x g for 5 min to get rid of dead cells, aliquoted and stored at −80°C. pLV[Exp]-EGFP:T2A:Hygro-hPGK> lentiviruses packaged with either N-tap-PGRN, GRN2+Linker 3 or GRN4+Linker 5, included the same coding region from AAV plasmids used for mouse neonatal injections and were produced by VectorBuilder, Inc.

#### Lentiviral transductions

Transduction of MEF *Grn*^+/+^ and *Grn*^−/−^ cells and selection was performed as previously described.^71^ Transduced MEF cell pools were maintained in DMEM, hi-FBS, and 1–2 μg/mL puromycin and subjected to clonal isolation. The clones were expanded and assessed for TMEM192_3xHA expression via western blot. The clones with robust TMEM-3xHA expression were chosen, expanded, and frozen. To generate PGRN or granulin add-back pools, PGRN_EGFP and GRN2L3_EGFP lentiviruses (VectorBuilder Inc.) were used to transduce TMEM192_3xHA-expressing MEF *Grn*^−/−^ (KOTH). The new KOTH lines stably expressing PGRN (KO-PGRN) and GRN2L3 (KO-GRN2) were maintained in DMEM plus 10% hi-FBS, 2 μg/mL puromycin and 200 μg/mL Hygromycin B and subjected to clonal enrichment. Isolated MEF cell lines were analyzed for expression of TMEM192–3xHA, PGRN, granulins by western blot The lines with high expression levels were chosen, expanded, and frozen.

#### Fluorescent immunocytochemistry

Triple-label fluorescent immunocytochemistry was performed to visualize lysosomal and mitochondrial co-localization of GRNs in PGRN, GRN2, GRN4-expressing TMEM192×3HA *Grn*^−/−^ MEF cells with lysosomal marker (CTSD), and a mitochondrial marker (HSP60). Cells were cultured as described above in growth medium and plated on Poly-L-Lysine coated 12mm coverglass in 24 well plates. Cells were washed twice with dPBS and fixed with 4% Paraformaldehyde for 20minutes at room temperature (RT). Proceeded with 3 times dPBS wash, and permeabilized with 0.1% Triton dPBS for 15 min at RT. Then, it was blocked with 5% normal donkey serum for 30 min and followed by incubation with optimal dilutions of antibodies mixture at 4^◦^ overnight with shaking. After three washes (10 min each) in dPBS, cells were incubated with optimal concentrations of fluorescent-labeled secondary antibodies and DAPI (1ug/ml) mixture for an hour. Bound primary antibodies were detected with Alexa Fluor 488 donkey anti-chicken IgG, Alexa Fluor 568-donkey anti-goat IgG, and Cy5-donkey anti-rabbit IgG. After three washes, sections were mounted, coverslipped with Immuno-mount fluorescent mounting media (Thermo Fisher) and imaged using Lecia STELLARIS SP8 microscope with a DFC9000 GT camera and system software (LAS X Life Science microscope software). 5 different regions of interests were imaged and analyzed using an automated pipeline created using CellProfiler (www.cellprofiler.org)^85^ for quantification.

#### Cell homogenization, Lyso-IP, and immunoblotting

Two 15 cm plates for each of the five lines stably expressing TMEM192_3xHA were seeded with 3 million cells in growth medium and grown for 24 h. Cells were washed twice with PBS, then gently scrapped with PBS/citric saline (135 mM KCl, 15 mM sodium citrate), after citric saline pre-treatment (1 min at 37°C), and spun at 1,000 xg for 1 min at 4°C. Pelleted cells were resuspended in 950 μL homogenization buffer (50 mM MES pH 6.5, 90 mM KCL, 6 mM magnesium acetate, 1× Halt protease and phosphatase inhibitor cocktail, 1 μL/mL endonuclease Denarase (c-LEcta GmbH).^89^ Cells were then loaded into 1-cc syringes through 20-gauge needles and slowly passed through a 16-μm gap in a cell homogenizer (Isobiotec) once in one direction, with uniform manual pressure. Proceeded to Lyso-IP as described by Abu-Remaileh et al., 2017 with modifications. 15 μL of the suspension was reserved for evaluation of cell homogenization quality by trypan blue exclusion, and the remainder centrifuged at 1,000 x g for 10 min at 4°C. The resulting pellet was reserved on ice for further protein extraction, while 90% of the light post-nuclear suspension (PNS), that contains mainly cytosol and small vesicles, was added to pre-washed anti-HA magnetic beads (ThermoFisher Scientific) and incubated on a MACmix rotator (Miltenyi Biotech) at room temperature (RT) for 5 min. Half of the remaining 10% of PNS was reserved on ice for immunoblotting, as Lyso-IP input. From here on all tubes used were low-retention tubes. The beads were gently rinsed by single resuspension in 1 mL of homogenization buffer and transferred to a new tube, three times. Beads were magnetically collected at the bottom of tube before the addition of 2 consecutive changes of lysis buffer (50 mM Tris pH 8.0, 150 mM NaCl, 1% Triton-Tx100, 2X Halt protease and phosphatase inhibitor cocktail) and incubated on ice for 10 min each time. Tubes were then placed on a magnetic rack and the supernatants were pooled into fresh tubes and kept on ice. Meanwhile, the remaining cytoplasmic/membrane proteins were extracted from the homogenate cell pellets to generate a cell lysate (C.L.). Pellets were resuspended in 950 μL of gentle lysis buffer (10 mM Tris pH 8.0, 15 mM NaCl, 5 mM magnesium acetate, plus 1× Halt protease and phosphatase inhibitor cocktail) and incubated on ice for 10 min. Triton Tx100 and CHAPS were added to 0.1% and 0.6% final concentrations, respectively, then tubes were inverted ten times before centrifugation at 1,000 x g at 4°C for 5 min. Supernatants were transferred to fresh tubes and pelleted nuclei were frozen. All supernatants were aliquoted and stored at −80°C for later use. Subsequently, an aliquot of each supernatant (cell lysate, input, and Lyso-IP samples) was thawed on ice and protein concentration was measured using Pierce BCA Protein Assay (ThermoFisher Scientific). SDS-PAGE samples normalized for protein concentration were made, reduced, and denatured, then loaded into 10% BisTris/MES NuPAGE gels (ThermoFisher Scientific) run and transferred to 0.22 μm-nitrocellulose membranes with a Transblot Turbo apparatus (Bio-Rad). Blots were then blocked for 1 h at RT, sectioned, incubated in primary antibodies at 4°C overnight and in secondary antibodies for 1 h at RT. Blot images were acquired on either an Odyssey Fc (ECL) or Odyssey M scanner (Li-Cor Biotechnology) and analyzed using Image Studio software (Li-Cor Biotechnology).

#### Flash frozen mouse brain sample processing for immunoblot

To prepare samples for the immunoblot analysis of proteins, a novel protocol was developed in which approximately 40 mg of mouse hippocampal tissue from each sample was placed in a solution of PBS with added HALT phosphatase protease inhibitor (PPI) at a dilution of 1:2 (weight to volume). The PPI was diluted into the 1xPBS at 1:100. In the PBS + PPI solution, the sample was cut into smaller pieces with mini scissors. Once cut into smaller pieces, the sample is ready for further homogenization.

A bead lysis kit was used for the homogenization of these small, soft hippocampal samples. The samples were cut into pieces and, still in the PBS + PPI solution, pipetted into 1.5 mL RINO screw-tap tubes (Next Advance) prefilled with zirconium oxide beads. Tubes were placed into the Bullet Blender (Next Advance) for homogenization.

Once homogenized, the solution was diluted 1:5 in RIPA lysis buffer supplemented with 1x HALT protease and phosphatase inhibitor. After 15 min, the solution was sonicated (30A; 2 s on; 8 s of rest; 10 s total sonication time/sample). After sonication, the solution was spun down in a centrifuge at 20,000xRCF at 4C for 10 min. Protein concentration was measured with the bicinchoninic acid (BCA) assay, samples were frozen in aliquots at −80C.

#### Immunoblot

SDS/PAGE and immunoblotting of HeLa cell lysates, cell media, and mouse brain lysates were performed as described.^26,84,96^ Mouse brain running samples were prepared for immunoblot in 1X Laemmli loading buffer with 20 mM tris(2-carboxyethyl)phosphine (TCEP)) followed by denaturation at 70°C for 15 min. For immunoblotting, protein samples were first separated on Bio-Rad TGX 4–20% 26-well gels at 100 V and transferred to a 0.2-μm nitrocellulose membrane using the Bio-Rad *Trans*-blot Turbo system. After BulletBlock (Nacalai) for 30 min at room temperature, membranes were incubated overnight at 4°C with primary antibodies (STAR MATERIALS). Membranes were probed with anti-Histone H3 or anti-Beta tubulin antibodies and imaged on the Odyssey Fc (LI-COR), to normalize protein abundance between samples.

For hGRN2 and hGRN4 protein samples were separated using 4–20% BisTris gels run using MES buffer (Genscript) at 100V to resolve bands. Transfers were completed using Bio-Rad *Trans*-blot Turbo to a 0.2-μm nitrocellulose membrane, then blocked with Fish Serum Blocking Buffer (ThermoFisher) for 60 min at room temperature. Membranes were then incubated with primary antibodies (1 μg/mL) overnight at 4C. All primary antibodies were diluted to a final concentration of 50% glycerol for long term storage at −20C. Near-infrared fluorescent secondary antibodies (diluted in TBST) or HRP-conjugated (diluted in 0.5% milk in TBST) antibodies (STAR Materials) were incubated for 1 h at room temperature. For HRP visualization, blots were incubated in Chemi-Lumi One Super (Nacalai) or SuperSignal West Femto (Thermo) for 5 min before imaging. Near infrared or chemiluminescent blots were imaged using Odyssey Fc (LI-COR) and analyzed by Image Studio software 5.2 (LI-COR).

#### Protein alignment and percent identity

Granulin 1–7 amino acid sequences were accessed from Uniprot Human: P28799). Sequences were aligned using the msa R package with ClustalOmega.^97^ Alignments were visualized and consensus sequence calculated using ggmsa.^98^ Percent Identity of amino acids was calculated from the ClustalOmega hGRN alignment using Bio3D.^87,88,90^

**Table:** 

Granulin	UniProt Accession number

hGRN1 (G)	PRO_0000012695
hGRN2 (F)	PRO_0000012696
hGRN3 (B)	PRO_0000012697
hGRN4 (A)	PRO_0000012698
hGRN5 (C)	PRO_0000012699
hGRN6 (D)	PRO_0000012700
hGRN7 (E)	PRO_0000012701

#### Diagrams

Some diagrams were made using BioRender (biorender.com).

### QUANTIFICATION AND STATISTICAL ANALYSIS

#### Proteomics, lipidomics, and metabolomics

LC/MS data was Log2 transformed and ANOVAs for the following comparisons were performed (GFP-*Grn*^−/−^ and GFP-*Grn*^*+/+*^) (GFP-*Grn*^−/−^ and hPGRN-*Grn*^−/−^*)* (GFP-*Grn*^−/−^ and hGRN2-*Grn*^−/−^*)* (GFP-*Grn*^−/−^ and hGRN4-*Grn*^−/−^) *p*-values were adjusted using the Benjamini-Hochberg method. Abundance of individual proteins of interest were analyzed using One-Way ANOVA followed by Tukey’s post-hoc analysis. Variance was assessed using the Brown-Forsythe test, *p* = 0.05 and the normality of GFP *Grn*^−/−^ and GFP-*Grn*^*+/+*^ samples was determined using the Shapiro-Wilk test *p* = 0.05. PCA confidence intervals were analyzed using the PCAtools R package, alpha set to 95%. The area ratios of endogenous lipids, metabolites, and surrogate internal standards were quantified using SCIEX OS 3.1. Statistical analysis of significance for lipid and metabolite levels in samples was determined by One-way ANOVA with Tukey’s post-hoc analysis. *p < 0.05, **p < 0.01, ***p < 0.001, ****p < 0.0001. (GFP-*Grn*^−/−^ and GFP-*Grn*^*+/+*^) (GFP-*Grn*^−/−^ and hPGRN-*Grn*^−/−^*)* (GFP-*Grn*^−/−^ and hGRN2-*Grn*^−/−^*)* (GFP-*Grn*^−/−^ and hGRN4-*Grn*^−/−^) comparisons are visualized in figures.

#### Immunohistochemistry and lipofuscin

IHC image quantification was performed single brain sections from 5 animals per group (*n* = 5), except for lipofuscin accumulation which was quantified from a single brain section from 9 to 13 animals per group (*n* = 9–13). Normality of GFP *Grn*^−/−^ and GFP-*Grn*^+/+^ samples was assessed using Shapiro-Wilk test *p* = 0.05 and variance was assessed using Brown-Forsythe test *p* = 0.05. Comparisons were conducted using two-way ANOVA, one factor being brain region and the second being AAV treatment group. A full effect model was fit and Tukey’s post-hoc analysis was completed comparing treatments groups to all other treatment groups within brain region.

#### Fluorescent immunocytochemistry

FICC image quantification was performed in 5 different regions of interest across 2 different coverslips per group in hGRNs-*Grn*^−/−^ MEF cells. Images were obtained by Leica SP8 Stellaris microscope with LAS X software and co-localization was assessed by Pearson’s correlation co-efficiency in CellProfiler software. Comparisons were conducted using two-way ANOVA, one factor being the different organelle markers and the second being the transduction group.

#### Western Blot and ELISA quantification

All blots were run using 5 individual animals per group (*n* = 5) and normalized values were analyzed using One-Way ANOVA followed by Tukey’s post-hoc analysis. Variance nd normality were assessed in the same manner as immunohistochemistry experiments. All regions were assessed independently. The hippocampal galectin-3 outlier was identified using Grubbs test *p* = 0.0001. ELISA data was analyzed using One-Way ANOVA followed by Tukey’s post-hoc test.

#### Visualization

All bar charts were produced in PRISM version 9 and heatmaps were made using Quickomics.^91^

## Supplementary Material

1

2

3

4

5

6

7

## Figures and Tables

**Table:** KEY RESOURCES TABLE

REAGENT or RESOURCE	SOURCE	IDENTIFIER

Antibodies		

Goat polyclonal anti-human progranulin	R and D Systems	Cat# AF2420; RRID: AB_2114489
Rabbit polyclonal anti-human granulin 2	This paper	N/A
Rabbit polyclonal anti-human granulin 4	This paper	N/A
Goat polyclonal anti-mouse galectin-3	R and D Systems	Cat# AF1197; RRID: AB_2234687
Goat polyclonal anti-mouse cathepsin Z	Thermo Fisher Scientific	PA5–47048; RRID: AB_2576470
Rabbit monoclonal anti-mouse CD68	Cell Signaling Technology	Cat# 97778; RRID: AB_2928056
Goat polyclonal anti-GFP	Rockland	Cat# 600–101-215; RRID: AB_218182
Rabbit polyclonal anti-Beta Tubulin	Cell Signaling Technology	Cat#2146; RRID: AB_2210545
Rabbit recombinant monoclonal Histone H3	Cell Signaling Technology	Cat#4499; RRID: AB_10544537
Chicken anti-MAP2	Neuromics	Cat# CH22103; RRID: AB_2314763
Goat polyclonal anti-AIF-1/Iba1	Novus	Cat# NB100–1028 RRID: AB_521594
Mouse monoclonal anti-Glial Fibrillary Acidic Protein (GFAP)	Sigma-Aldrich	Cat# G3893 RRID: AB_477010
Chicken anti-HSP60	EnCor Biotechnology	Cat#CPCA-HSP60 RRID: AB_2572330
Mouse monoclonal anti-Strep-TagII (C23.21)	Absolute Antibody	Ab02208–1.1
Strep-TactinXT-HRP	IBA Lifesciences	custom
Goat anti-rabbit IgG (H + L) DyLight800	Cell Signaling Technology	Cat# 5151; RRID: AB_10697505
Goat anti-mouse IgG IRDye 680RD	LI-COR Biosciences	Cat#925–68070 RRID:AB_2651128
Donkey anti-goat IgG (H + L) Cross-Adsorbed Secondary Antibody, Alexa Fluor 568	Thermo Fisher Scientific	Cat# A-11057 RRID: AB_2534104
Donkey anti-chicken IgY (IgG) (H + L), Alexa Fluor 488	Jackson ImmunoResearch Labs	Cat# 703–545−155; RRID: AB_2340375
Donkey anti-mouse IgG (H + L) Antibody, Alexa Fluor 488 Conjugated	Thermo Fisher Scientific	Cat# A21202 RRID: AB_141607
Donkey anti-Rabbit Cy5-AffiniPure	Jackson ImmunoResearch Labs	Cat# 711–175-152 RRID: AB_2340607
Goat anti-Rabbit HRP	Cell Signaling Technology	Cat#7074P2; RRID: AB_2099233
Donkey anti-Goat IgG (H + L) Highly Cross-Adsorbed Secondary Antibody, Alexa Fluor Plus 800	Thermo Fisher Scientific	Cat#A32930; RRID: AB_2762842
Donkey anti-Rabbit IgG (H + L) Highly Cross-Adsorbed Secondary Antibody, Alexa Fluor Plus 680	Thermo Fisher Scientific	Cat#A32802; RRID: AB_2762836
Biotinylated horse anti-rabbit IgG (H + L)	Vector Laboratories	Cat#: BA-1100–1.5; RRID: AB_2336201
Biotinylated horse anti-goat IgG (H + L)	Vector Laboratories	Cat#: BA-9500–1.5; RRID: AB_2336123
Biotinylated rabbit anti-sheep immunoglobulin	Vector Laboratories	Cat#: BA-6000–1.5 RRID: AB_2336217
Bacterial and virus strains		
Lentivirus pLJC5-Tmem192–3xHA	This paper	RRID:Addgene_102930
Lentivirus pLV[Exp]-EGFP:T2A: Hygro-hPGK>{N-TAP PGRN}	VectorBuilder	Catalog #: LVS(VB190712–1034dkb)-C
Lentivirus pLV[Exp]-EGFP:T2A: Hygro-hPGK>{GRN2+linker}	VectorBuilder	Catalog #: LVS(VB190712–1032cbw)-C
Lentivirus pLV[Exp]-EGFP:T2A: Hygro-hPGK>{GRN4+linker}	VectorBuilder	Catalog #: LVS(VB190712–1030aad)-C
rAAV1 strep-GFP	This Paper	Addgene; cat # in progress
rAAV1 hPGRN	This Paper	Addgene; cat # in progress
rAAV1 hGRN4	This Paper	Addgene; cat # in progress
rAAV1 hGRN2	This Paper	Addgene; cat # in progress
Chemicals, peptides, and recombinant proteins		
DAPI Nucleic Acid Stain	Thermo Fisher Scientific	Cat# 62248; CAS: 28718–90-3
Pink Rino Tubes	Next Advance	Cat# PINKR1-RNA
Bullet Block	Nacalai	Cat# 13779–01
HALT Protease and phosphatase inhibitor	Thermo Fisher Scientific	Cat# UK286007
Diaminobenzidine (DAB)	TCI-Chemicals	Cat# D0077
Pertex Mounting medium	CellPath	Cat# SEA-0100–00A
Epredia Immu-Mount	Fisher Scientific	Cat# 9990402
SuperSignal West Femto Maximum Sensitivity Substrate	Thermo Fisher Scientific	Cat# 34095
Opti-MeM Reduced Serum Media	Gibco	Cat# 31985070
DMEM high glucose, pyruvate	Gibco	Cat# 11995073
Benzonase^®^	Sigma	Cat# E8263–25KU
HiTrap Q HP column	Amersham Biosciences	Cat# 17115401
Lysyl endopeptidase	WAKO	Cat# 125–05061
Trypsin	Pierce	Cat# 90059
Sep-Pak C18 column	Waters	Cat# WAT036945
18-plex Tandem Mass Tag (TMTPro) kit	Thermo Fisher Scientific	Cat# A44520 and A52046
60mg HLB column	Waters	Cat# WAT094226
BEH column	Waters	Cat# 186002353
ReproSil-Pur: 120 C18-AQ 1.9um	Dr. Maisch HPLC GmbH	Cat# r119.aq.0001
ACQUITY Premier BEH C18 Column, 1.7 μm, 2.1 × 100 mm	Waters	Cat# 186009453
ACQUITY Premier BEH Amide Column, 1.7 μm, 2.1 mm × 150 mm	Waters	Cat#186009506
HALO HILIC 2.0 μm, 3.0 × 150 mm column	Advanced Materials Technology	Cat#PN 91813–701
REVERT total Protein Stain	LI-COR	Cat#926–11010
Fish Serum Blocking Buffer	Thermo Fisher Scientific	Cat#37527
Lysing Matrix D tubes	MP Biomedicals	Cat#116913050
MES Running Buffer	Genscript	Cat#M00677
ZymoPURE II Plasmid Maxiprep kit	Zymo Research	Cat#Z5139
TransIT-LT1 Transfection reagent	Mirus Bio	Cat#MIR 2300
Denarase	c-LEcta	Cat#VWR-20804–100K
Anti-HA magnetic beads	Thermo Fisher Scientific	Cat#88836
Critical commercial assays		
Mouse on Mouse ImmPress HRP Polymer kit	Vector Laboratories	Cat#MP-2400
Avidin-biotin-peroxidase complex (ABC)	Vector Laboratories	Cat#PK-6100
Mouse GPNMB ELISA	R and D Systems	Cat#DY2330
Human PGRN ELISA	R and D Systems	Cat#DY2420
Deposited data		
Mouse brain proteomic dataset	This paper; https://www.ebi.ac.uk/pride	PXD041095
Mouse brain metabolomics and lipidomics dataset	This paper; https://dataverse.unc.edu/dataverse/Emory	https://doi.org/10.15139/S3/7Z2SVL
Experimental models: Cell lines		
HeLa *Grn*^*+/+*^	Nguyen et al.^84^	N/A
HeLa *Grn*^*−/−*^	Nguyen et al.^84^	N/A
MEF *Grn*^*−/−*^	Nguyen et al.^84^	N/A
MEF *Grn*^*+/+*^	Nguyen et al.^84^	N/A
HEK293T	ATCC	Cat# CRL-3216; RRID:CVCL_0063
Experimental models: Organisms/strains		
Top10 *E coli*	Invitrogen	Cat#C404010
B6(Cg)-Grntm1.1Aidi	Jackson Labs	IMSR Cat# JAX:013175; RRID: IMSR_JAX:013175
Recombinant DNA		
pLV[Exp]-EGFP:T2A:Hygro-hPGK>{N-TAP PGRN}	VectorBuilder	Catalog #: Ecoli(VB190712–1034dkb)
pLV[Exp]-EGFP:T2A:Hygro-hPGK>{GRN2+linker}	VectorBuilder	Catalog #: Ecoli(VB190712–1032cbw)
pLV[Exp]-EGFP:T2A:Hygro-hPGK>{GRN4+linker}	VectorBuilder	Catalog #: Ecoli(VB190712–1030aad)
pLJC5-Tmem192–3xHA	Addgene	RRID: Addgene_102930
pCMV-VSV-G	Addgene	RRID: Addgene_8454
pCMV-dR8.2 dvpr	Addgene	RRID: Addgene_8455
pAAV strep-GFP	This Paper	Addgene; cat # in progress
pAAV hPGRN	This Paper	Addgene; cat # in progress
pAAV hGRN4	This Paper	Addgene; cat # in progress
pAAV hGRN2	This Paper	Addgene; cat # in progress
Software and algorithms		
Proteome Discoverer Suite (v.2.4.1.15)	Thermo Fisher Scientific	RRID:SCR_014477
Prism 9	Graphpad Software	https://www.graphpad.com/scientificsoftware/prism/; RRID:SCR_002798
IMARIS 10.0.1	Oxford Instruments	https://imaris.oxinst.com/support/imarisrelease-notes/10-0-0; RRID:SCR_007370
BioRender	https://www.biorender.com	RRID:SCR_018361
PCAtools R package	Blighe et al.^84^	https://github.com/kevinblighe/PCAtools
Metascape	Zhou et al.^85^	https://metascape.org; RRID:SCR_016620
Quickomics	Gao et al.^86^	https://quickomics.bxgenomics.com/
Ggmsa R package	Zhou et al.^87^	https://github.com/YuLab-SMU/ggmsa
Bio3D R package	Grant et al.^88^	http://thegrantlab.org/bio3d/; RRID:SCR_024266
CellProfiler	Lamprecht et al.^89^	www.cellprofiler.org;RRID:SCR_007358
Msa R	Bodenhofer et al.^90^	https://github.com/UBod/msa
Ggplot2	Wickham^91^	https://ggplot2.tidyverse.org; RRID:SCR_014601
SCIEX OS 3.1	Sciex	https://sciex.com/br/products/software/sciex-os-software
Microsoft R Open v3.4.2	Microsoft	https://mran.microsoft.com
LI-COR Image Studio	LI-COR	RRID:SCR_015795
Adobe Illustrator	Adobe	RRID:SCR_010279
Adobe Photoshop	Adobe	RRID:SCR_014199

## Highlights

- *Grn*^*−/−*^ mouse brain proteomics reveals inflammation and defects in lipids and lysosomes
- Expression of granulins using AAV ameliorates dysregulation of the thalamic proteome
- Granulins correct lysosome defects, inflammation, and lipofuscin in *Grn*^−/−^ mouse brain
- Granulins localize to lysosomes in *Grn*^−/−^ brain and fibroblasts, likely mediating activity
