# Supplementary material for: Granulins rescue inflammation, lysosome dysfunction, lipofuscin, and neuropathology in a mouse model of progranulin deficiency
Source: Cell Rep. Author manuscript; Available in PMC 2025 Jan 28. (PMC11773623; doi:10.1016/j.celrep.2024.114985)
Supplement: 1 [file NIHMS2044434-supplement-1.pdf]

Cell Reports, Volume *43*

Supplemental information

Granulins rescue inflammation, lysosome dysfunction, lipofuscin, and neuropathology in a mouse model of progranulin deficiency

Jessica Root, Anarmaa Mendsaikhan, Georgia Taylor, Paola Merino, Srijita Nandy, Minzheng Wang, Ludmilla Troiano Araujo, Danny Ryu, Christopher Holler, Bonne M. Thompson, Giuseppe Astarita, Jean-François Blain, and Thomas Kukar

A.

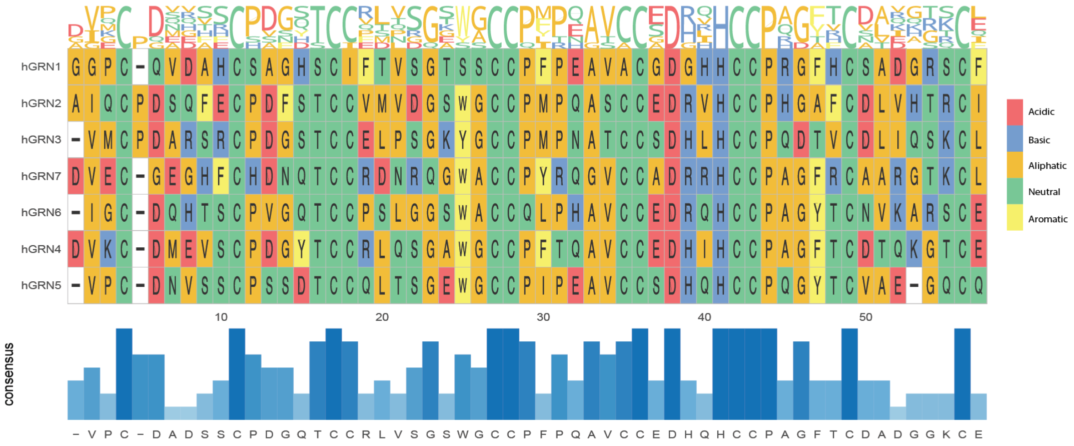

B.

Table 1	hGRN5	hGRN2	hGRN3	hGRN4	hGRN6	hGRN1	hGRN7
**hGRN5**	100.0%	46.3%	55.6%	59.3%	53.7%	40.7%	38.9%
**hGRN2**	46.3%	100.0%	51.8%	50.0%	49.1%	33.9%	41.1%
**hGRN3**	55.6%	51.8%	100.0%	52.7%	40.0%	34.5%	38.2%
**hGRN4**	59.3%	50.0%	52.7%	100.0%	52.7%	39.3%	48.2%
**hGRN6**	53.7%	49.1%	40.0%	52.7%	100.0%	38.2%	41.8%
**hGRN1**	40.7%	33.9%	34.5%	39.3%	38.2%	100.0%	33.9%
**hGRN7**	38.9%	41.1%	38.2%	48.2%	41.8%	33.9%	100.0%

Figure S1: Granulin peptide alignments and percent identity table, related to Figure 1.

A) ClustalW alignment of human granulin sequences. Visualized using ggmsa R package. Amino acids are color coded by their chemical properties.
B) Percent identity table derived from sequence similarity values calculated using granulin alignment output for figure S1A.

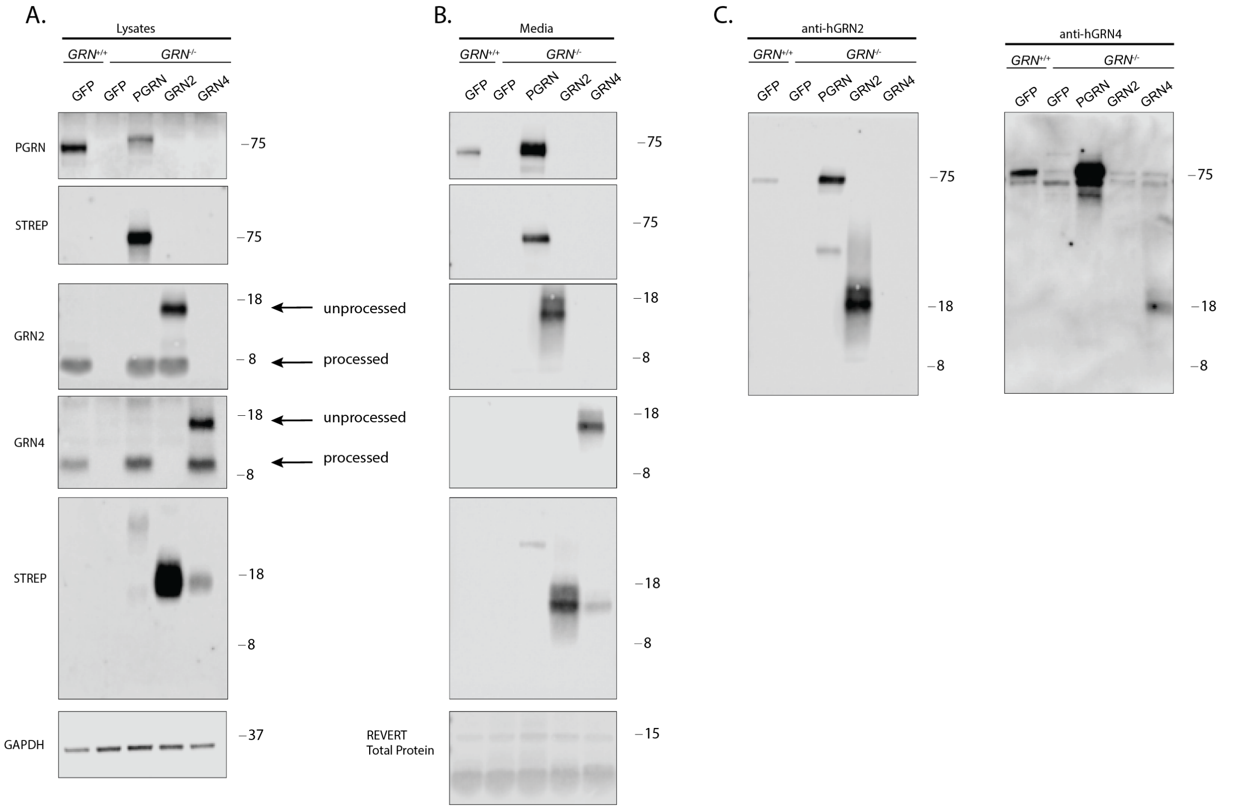

Figure S2: Validation that hPGRN, hGRN2, and hGRN4 are properly trafficked to the lysosome and are secreted, related to Figure 1.

A) Immunoblot of cell lysate from HeLa $GRN^{-/-}$ or $GRN^{+/+}$ cells transiently expressing hPGRN, hGRN2, or hGRN4. Probed for hPGRN, hGRN2, or hGRN4. 15 kDa band of hGRN transfected lysates is both hGRN and STREP positive and labeled as unprocessed. The 6 kDa band in hPGRN and hGRN2 transfected cell lysates is GRN2 positive, but not STREP positive, and labeled as the processed, cleaved granulin-2 peptide. The 8 kDa band in hPGRN and hGRN4 transfected cell lysates is GRN4 positive, but not STREP positive, and labeled as the processed, cleaved granulin-4 peptide.

B) Immunoblot of conditioned media collected from HeLa $GRN^{-/-}$ or $GRN^{+/+}$ cells expressing hPGRN, hGRN2, or hGRN4. Probed for hPGRN, hGRN2, or hGRN4. The 15 kDa band of hGRN2/4 transfected lysates is both hGRN and STREP positive and labeled as unprocessed. Mature processed GRNs are not observed in conditioned media.

C) Immunoblot of conditioned media collected from HeLa $GRN^{-/-}$ or $GRN^{+/+}$ cells expressing hGRN2 or hGRN4. Antibodies generated by the Kukar lab detect full length hPGRN (75kDa) and hGRN2 or hGRN4 (18 kDa), respectively.

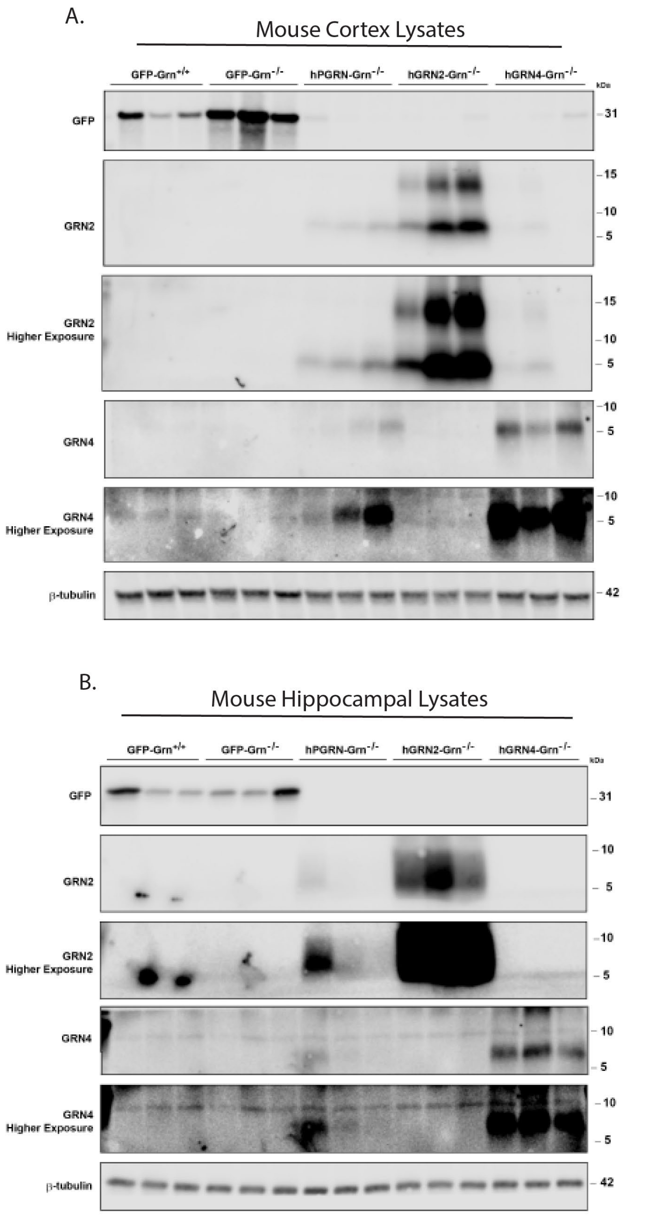

Figure S3: hPGRN, hGRN2, and hGRN4 are detected in rAAV injected mouse brain, related to Figure 1.

A) Immunoblot verifying expression of AAV delivered proteins following rAAV injection and aging. Cortical lysates were probed for GFP, hPGRN, hGRN2, hGRN4, and b-tubulin loading control. Additional images of hGRN signals after higher exposure of immunoblots reveal that hGRNs can be detected in hPGRN injected animals.
B) Immunoblots verifying expression of AAV delivered proteins following rAAV injection and aging in hippocampal lysates. Lysates were probed for GFP, hPGRN, hGRN2, hGRN4, and b-tubulin loading control. Additional images of hGRN signals after higher exposure reveal that hGRNs can be detected in hPGRN injected animals.

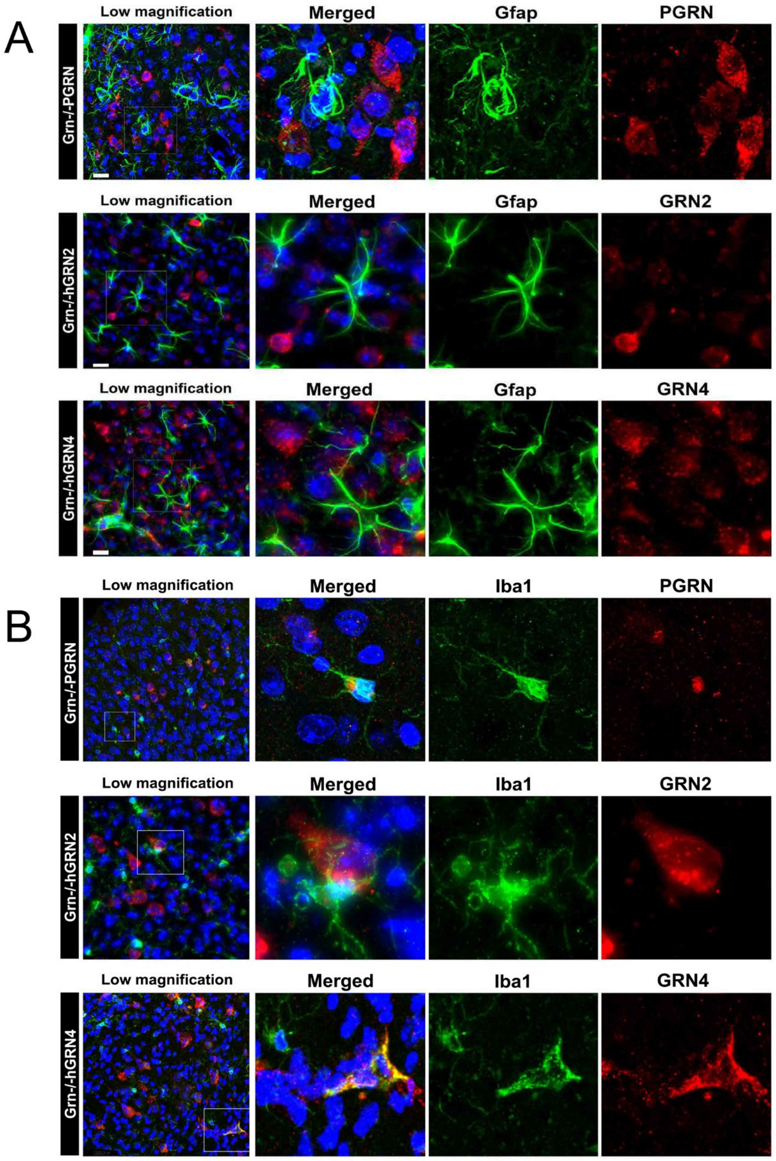

Figure S4: hPGRN, hGRN2, and hGRN4 with cellular markers, related to Figure 1.

A) Representative images from immunohistochemical staining showing localization of hGRNs and astrocyte marker Gfap in hPGRN, hGRN2, and hGRN4 expressing *GRN*$^{-/-}$ mice brain sections.
B) Representative images from immunohistochemical staining showing localization of hGRNs and microglial marker Iba1 in hPGRN, hGRN2, and hGRN4 expressing *GRN*$^{-/-}$ mice brain sections.

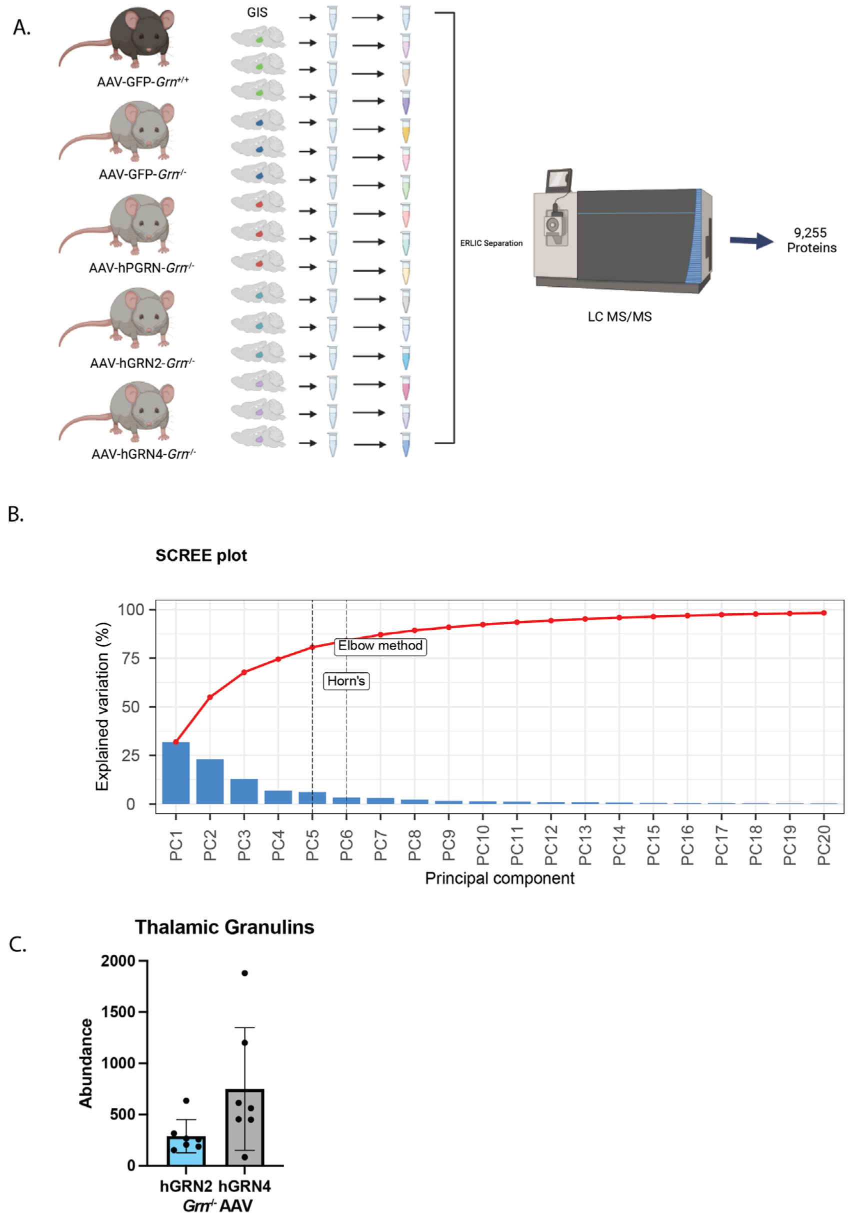

Figure S5: Thalamic Proteomics, related to Figure 2.

A) Diagram of the proteomic thalamic workflow, displaying the number of proteins detected, 9,255.
B) Assessment of Horn's Parallel Analysis to determine how many components of the PCA to retain in downstream consideration.
C) Welch's T-test comparing the abundance of granulin peptide detected in hGRN2-*Grn*$^{-/-}$ and hGRN4-*Grn*$^{-/-}$ mouse thalamus (p-value=0.091) mean hGRN2=288.4, mean hGRN4=749.3.

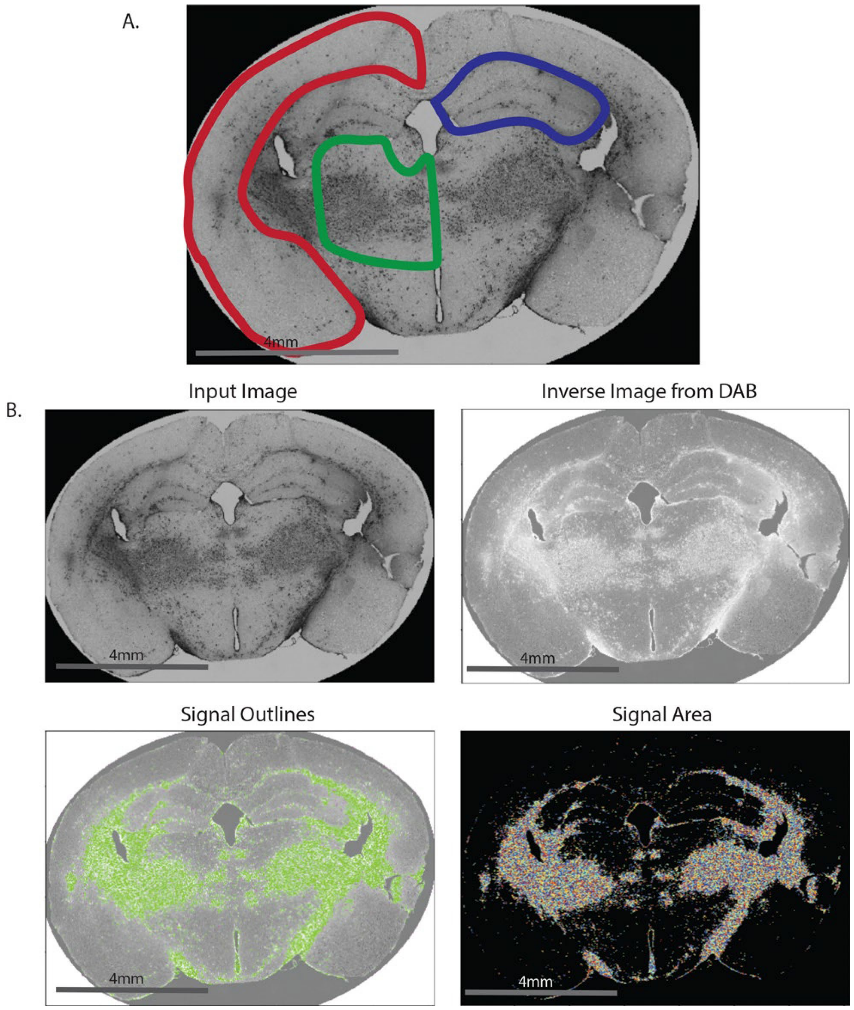

Figure S6: Cell Profiler Workflow, related to Star Methods.

A) Example of cropped ROIs extracted from whole coronal section images. Bilateral images were collected from each section for each region and used as input for CellProfiler quantification. Regions of interest are cortex (red), hippocampus (blue), and thalamus (green).
B) Overview of CellProfiler workflow and output. Signal Area represents the signal quantified in statistical analysis.

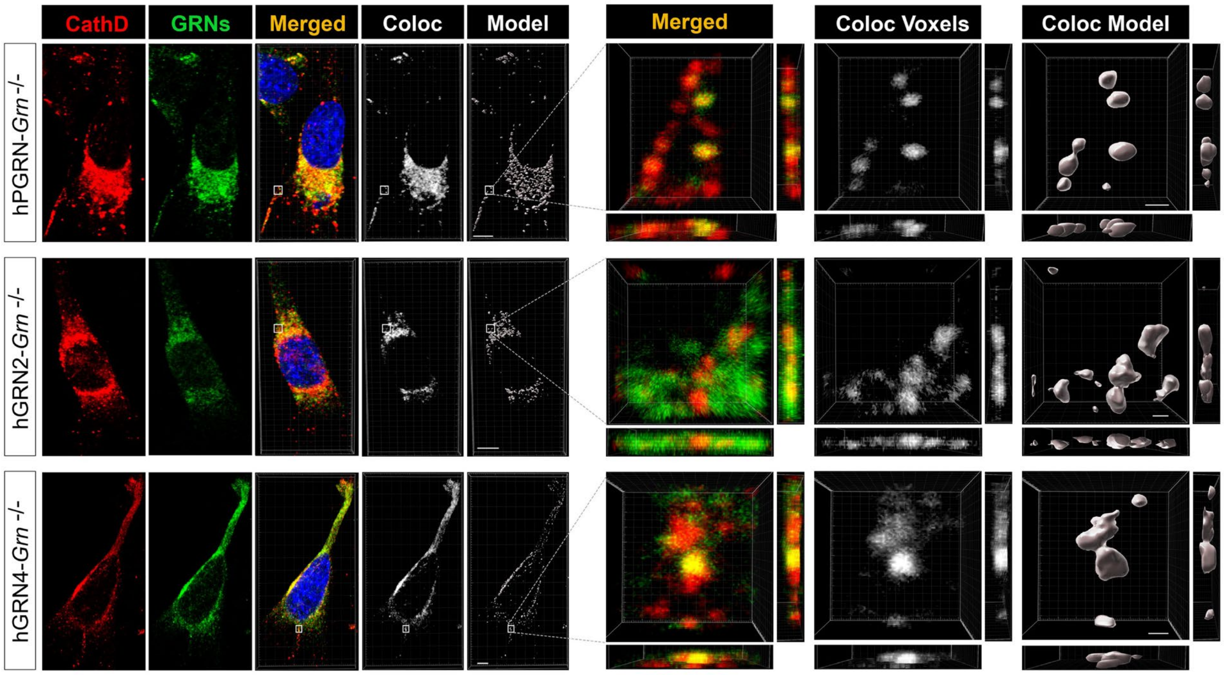

Figure S7: Granulins co-localize with CSTD-positive lysosomes in mouse embryonic fibroblasts expressing hPGRN, hGRN2, or hGRN4, related to Figure 6.

Fluorescent immunocytochemistry was performed on MEF *Grn*$^{-/-}$ TMEM192 3xHA cells expressing hPGRN, hGRN2, or hGRN4 to stain for the lysosomal protein cathepsin D (CathD; red), hGRNs (PGRN, hGRN2, or hGRN4; green), and nucleus (DAPI stain; blue). Scale bar = 5 µm. Co-localization of signals for CathD and granulins appears as yellow in merged channel. Images were analyzed with IMARIS software to identify specific voxels of CathD and GRNs that co-localize (coloc; white). A 3-dimensional model (coloc model) was built in IMARIS of co-localized CathD and GRNs fluorescent voxels. Higher magnification of boxed area (white box) is shown on the right to provide better visualization of merged fluorescent signal CathD and GRNs with colocalized voxels (coloc voxels) highlighted in white. Scale bar = 0.5 µm.

Table S1. DNA sequence and translated amino acid sequence of proteins encoded in pAAV plasmid, related to Figure 1 and STAR Methods

Plasmid name	DNA coding sequence	Amino acid sequence of encoded protein
**pAAV GFP**	atggcggccgccggctggagccaccctcagttcgagaagggaggaggaggcggagg<br>cggaggatggagccacccgcagttcgagaaaggagctagtggcggagagaatctgta<br>ttttcagggcggaggcggagctagcaagggcgaggagctgttcaccggggtggtgccc<br>atcctggtcgagctggacggcgacgtaaacggccacaagttcagcgtgtccggcgagg<br>gcgagggcgatgccacctacggcaagctgaccctgaagttcatctgcaccaccggcaa<br>gctgcccgtgccctggcccacactagtgaccaccctgtgctacggcgtgcagtgcttcag<br>ccgctaccccgaccacatgaagcagcacgacttcttcaagtccgccatgcccgaaggct<br>acgtccaggagcgcaccatcttcttcaaggacgacggcaacttcaagacccgcgccga<br>ggtgaagttcgagggcgacaccctggtgaaccgcatcgagctgaagggcatcgacttc<br>aaggaggacggcaacatcctggggcacaagctggagtacaactacaacagccacaa<br>cgtctatatcatggccgacaagcagaagaacggcatcaaggttaacttcaagacccgc<br>cacaacatcgaggacggcagcgtgcagctcgccgaccactaccagcagaacacccc<br>catcggcgacggccccgtgctgctgcccgacaaccactacctgagcacccagtccgcc<br>ctgagcaaagaccccaacgagaagcgcgatcacatggtcctgctggagttcgtgaccg<br>ccgccggcatcactcacggcatggacgagctgtacaagtaa	MAAAGWSHPQFEKG<br>GGGGGGGWSHPQFE<br>KGASGGENLYFQGG<br>GGASKGEELFTGVVPI<br>LVELDGDVNGHKFSV<br>SGEGEGDATYGKLTL<br>KFICTTGKLPVPWPTL<br>VTTLCYGVQCFSRYP<br>DHMKQHDFFKSAMPE<br>GYVQERTIFFKDDGN<br>FKTRAEVKFEGDTLV<br>NRIELKGIDFKEDGNIL<br>GHKLEYNYNSHNVYI<br>MADKQKNGIKVNFKT<br>RHNIEDGSVQLADHY<br>QQNTPIGDGPVLLPD<br>NHYLSTQSALSKDPN<br>EKRDHMVLLEFVTAA<br>GITHGMDELYK
**pAAV hPGRN**	atgtggaccctggtgtcctgggtggccctgacagccggactggtggccggatctgcctgg<br>tcccacccccagtttgagaagggcggaggctctggcggcggaagcggaggatctgctt<br>ggagccaccctcagttcgaaaagggggccagcggcaagcccatccccaatcctctgct<br>gggcctggacagcaccgagaacctgtattttcaaggcacccggtgtcccgacggccagt<br>tttgccctgtggcctgctgcctggaccctggcggagccagctacagctgctgcagacccct<br>gctggacaagtggcccaccaccctgagcagacacctgggcggaccttgccaggtgga<br>cgcccactgttctgccggccacagctgcatcttcaccgtgtccggcacctccagctgctgc<br>ccatttcctgaggccgtggcctgtggcgacggacaccactgctgccctagaggcttccac<br>tgcagcgccgacggcagaagctgcttccagcggagcggcaacaacagcgtgggcgc<br>catccagtgccccgactcccagttcgagtgccccgatttcagcacctgttgcgtgatggtg<br>gacggcagctggggctgctgtcccatgcctcaggccagctgttgcgaggaccgggtgca<br>ctgttgccctcacggcgccttttgcgacctggtgcacacccggtgcatcacccctaccggc<br>acccaccctctggccaagaaactgcccgcccagcggaccaacagagccgtggccctg<br>tctagcagcgtgatgtgccccgatgccagaagccgctgccctgacggcagcacatgctg<br>cgagctgcccagcgggaagtacggctgttgcccaatgcccaacgccacctgttgcagc<br>gaccatctgcactgctgtccacaagacaccgtgtgcgacctgatccagagcaagtgcct<br>gagcaaagagaacgccaccaccgacctgctgaccaagctgcctgcccacaccgtgg<br>gcgacgtgaagtgcgacatggaagtgtcctgccccgatggctacacctgttgccggctgc<br>agagcggcgcctggggatgctgccctttcacccaggccgtgtgttgtgaggaccacatcc<br>attgctgccctgccggcttcacctgtgacacccagaagggaacctgcgagcagggccc<br>acaccaggtgccctggatggaaaaggcccctgcccacctgagcctgcccgatcctcag<br>gccctgaagagggacgtgccctgcgacaacgtgtccagctgccccagcagcgatacct<br>gctgccagctgacatccggcgagtggggctgttgtcctatccccgaagccgtgtgctgctc<br>cgatcaccagcactgttgtcctcaaggctacacatgcgtggccgagggccagtgtcagc<br>ggggatctgagatcgtggccggcctggaaaagatgcccgccagaagggccagcctga<br>gccaccccagagacatcggctgcgaccagcacacctcctgccctgtgggccagacttg<br>ctgtcctagcctgggcggctcctgggcctgctgtcagctgcctcacgctgtgtgctgtgagg<br>ataggcagcattgctgtcccgccggatacacatgcaacgtgaaggcccggtcctgcgag<br>aaagaagtggtgtccgcccagcccgccaccttcctggccagatctccacacgtgggagt<br>gaaggacgtggaatgcggcgagggccacttctgccacgacaaccagacatgctgccg<br>ggacaaccgccagggctgggcctgttgcccatacagacagggcgtgtgctgtgctgatc	MWTLVSWVALTAGLV<br>AGSAWSHPQFEKGG<br>GSGGGSGGSAWSHP<br>QFEKGASGKPIPNPLL<br>GLDSTENLYFQGTRC<br>PDGQFCPVACCLDPG<br>GASYSCCRPLLDKWP<br>TTLSRHLGGPCQVDA<br>HCSAGHSCIFTVSGTS<br>SCCPFPEAVACGDGH<br>HCCPRGFHCSADGR<br>SCFQRSGNNSVGAIQ<br>CPDSQFECPDFSTCC<br>VMVDGSWGCCPMPQ<br>ASCCEDRVHCCPHGA<br>FCDLVHTRCITPTGTH<br>PLAKKLPAQRTNRAV<br>ALSSSVMCPDARSRC<br>PDGSTCCELPSGKYG<br>CCPMPNATCCSDHLH<br>CCPQDTVCDLIQSKCL<br>SKENATTDLLTKLPAH<br>TVGDVKCDMEVSCPD<br>GYTCCRLQSGAWGC<br>CPFTQAVCCEDHIHC<br>CPAGFTCDTQKGTCE<br>QGPHQVPWMEKAPA<br>HLSLPDPQALKRDVP<br>CDNVSSCPSSDTCCQ<br>LTSGEWGCCPIPEAV<br>CCSDHQHCCPQGYT<br>CVAEGQCQRGSEIVA

	ggcgccactgttgcccagccggctttagatgcgccgccaggggcaccaagtgtctgcgg agagaagcccccagatgggacgccccccctgagagatcccgccctgagacagctgctg	GLEKMPARRASLSHP RDIGCDQHTSCPVGQ TCCPSLGGSWACCQL PHAVCCEDRQHCCPA GYTCNVKARSCEKEV VSAQPATFLARSPHV GVKDVECGEGHFCH DNQTCCRDNRQGWA CCPYRQGVCCADRR HCCPAGFRCAARGTK CLRREAPRWDAPLRD PALRQLL
**pAAV hGRN2**	atgtggaccctggtgtcctgggtcgcactgacagcaggactggtggctggatctgcatgg agtcacccccagttcgagaagggaggaggatccggaggaggatcaggagggagcg catggtcccatcctcagtttgaaaaaggcgcctctgactacaaggacgatgacgataaa gctatccagtgccctgactctcagttcgagtgtccagacttcagcacatgctgcgtgatggt cgatggatcatggggctgctgtcccatgcctcaggccagctgctgtgaagacagggtgc actgctgtccacatggcgccttctgcgatctggtccacactcggtgtattaccccaacagg gactcatcccctggccaagaaactgccagctcagcgaaccaacagagcagtggctctg agctcctgac	MWTLVSWVALTAGLV AGSAWSHPQFEKGG GSGGGSGGSAWSHP QFEKGASDYKDDDDK AIQCPDSQFECPDFST CCVMVDGSWGCCPM PQASCCEDRVHCCPH GAFCDLVHTRCITPTG THPLAKKLPAQRTNR AVALSS
**pAAV hGRN4**	atgtggactctggtgtcctgggtcgcactgaccgcaggactggtggctggaagcgcatgg tcccacccacagttcgagaagggaggaggatctggaggaggatctggagggagtgca tggtcacatcctcagtttgagaagggagccagtgactacaaagacgatgacgataagg acgtgaaatgcgatatggaagtcagctgtccagatgggtatacatgctgtcgactgcagt ccggagcatggggatgctgtcctttcactcaggccgtgtgctgtgaagaccacatccattg ctgtccagctggctttacctgcgatacacagaaaggcacctgtgagcaggggccacacc aggtcccctggatggaaaaggcaccagctcatctgagcctgccagaccctcaggcccct gaaaagatga	MWTLVSWVALTAGLV AGSAWSHPQFEKGG GSGGGSGGSAWSHP QFEKGASDYKDDDDK DVKCDMEVSCPDGYT CCRLQSGAWGCCPF TQAVCCEDHIHCCPA GFTCDTQKGTCEQGP HQVPWMEKAPAHLSL PDPQALKR
